# Metagenomic characterization of bacterial community and antibiotic resistance genes in representative ready-to-eat food in southern China

**DOI:** 10.1038/s41598-020-72620-4

**Published:** 2020-10-22

**Authors:** YiMing Li, WeiWei Cao, ShuLi Liang, Shinji Yamasaki, Xun Chen, Lei Shi, Lei Ye

**Affiliations:** 1 College of Light Industry and Food Sciences, South China University of Technology, Guangzhou, 510640 China; 2 Institute of Food Safety and Nutrition, Jinan University, Guangzhou, 510632 China; 3 School of Bioscience and Bioengineering, South China University of Technology, Guangzhou, 510006 China; 4 Graduate School of Life and Environmental Sciences, Osaka Prefecture University, Osaka, 140-0002 Japan

**Keywords:** Metagenomics, Microbiome

## Abstract

Ready-to-eat (RTE) foods have been considered to be reservoirs of antibiotic resistance bacteria, which constitute direct threat to human health, but the potential microbiological risks of RTE foods remain largely unexplored. In this study, the metagenomic approach was employed to characterize the comprehensive profiles of bacterial community and antibiotic resistance gene (ARG) in 18 RTE food samples (8 RTE meat, 7 RTE vegetables and 3 RTE fruit) in southern China. In total, the most abundant phyla in RTE foods were Proteobacteria, Firmicutes, Cyanobacteria, Bacteroidetes and Actinobacteria. 204 ARG subtypes belonging to 18 ARG types were detected with an abundance range between 2.81 × 10^−5^ and 7.7 × 10^−1^ copy of ARG per copy of 16S rRNA gene. Multidrug-resistant genes were the most predominant ARG type in the RTE foods. Chloramphenicol, macrolide-lincosamide-streptogramin, multidrug resistance, aminoglycoside, bacitracin, tetracycline and β-lactam resistance genes were dominant, which were also associated with antibiotics used extensively in human medicine or veterinary medicine/promoters. Variation partitioning analysis indicated that the join effect of bacterial community and mobile genetic elements (MGEs) played an important role in the resistome alteration. This study further deepens the comprehensive understanding of antibiotic resistome and the correlations among the antibiotic resistome, microbiota, and MGEs in the RTE foods.

## Introduction

Due to the overuse and misuse of antibiotics in agricultural, livestock breeding and human medical environments, the problem of antibiotic resistant (AR) bacteria is growing, and constitutes a real threat to public health^1,2^. Many environments, such as soil, sludge, surface water and animal waste, have been proven to be important reservoirs for antibiotic resistance genes (ARGs) because many ARGs have been detected in these environments^3,4^. Recent studies have found that food (meat, vegetables and fruit) not only serve as a reservoir of ARGs and AR bacteria but also as a mediator to transfer ARGs and AR bacteria between the environment and humans by direct contact or indirectly through the consumption of contaminated foods^5,6^. Humans consume foods containing AR bacteria, and the exchange of ARGs between bacteria from food and human intestinal microorganisms may lead to the accumulation of ARGs in humans, which may affect the efficacy of antibiotics^7,8^. Consequently, in-depth investigations of the diversity and abundance of bacterial community and ARGs in food are central to establishing the overall picture and essential for management decision frameworks aimed at controlling antibiotic resistance.

Food contains a wide range of microorganisms, including AR bacteria^5,9^. Ready-to-eat (RTE) foods are not intended to undergo a serve heating step before consumption; hence, RTE foods have been recognized as potential vehicles of microbial food-borne bacteria^10^. ARGs present in RTE foods can be transmitted to bacteria in the human gut by horizontal gene transfer through mobile genetic elements (MGEs) such as plasmids, transposons and integrons^11^. This constitutes a direct threat to human health and necessitates more public concern worldwide. Current studies have mainly focused on the prevalence, antimicrobial resistance and genetic diversity of the food-borne pathogens, such as *Escherichia coli*^12,13^, *Salmonella*^14^, and *Staphylococcus aureus*^15^. Guo et al. revealed that out of 99 *Escherichia coli* isolates, 24.2% (24/99) were resistant to at least one antimicrobial agent in RTE food in Singapore^12^. Yang et al. observed that the prevalence of *Salmonella* was 9% among the 221 RTE food samples from Shanghai and twenty isolates were resistant to trimethoprim/sulfamethoxazole and sulfisoxazole^14^. Yang et al. reported that 75.8% (47/62) of the methicillin-susceptible *Staphylococcus aureus* (MRSA) were isolated from retail RTE foods in China and all of the MRSA isolates were resistant to three or more antibiotics^15^.

However, due to the limitations of traditional methods of microbiological isolation culture, a large number of microbiota cannot be isolated^16,17^. Thus, we cannot fully understand the distribution of AR bacteria in food using such methods. Although many molecular technologies, such as polymerase chain reaction (PCR)^18^, quantitative PCR (qPCR)^19,20^ and droplet digital PCR (ddPCR)^21^ have been reported to determine the distribution and occurrence of ARGs in food-borne bacteria, these methods also suffer from several limitations, including a limited number of validated primers for targeted genes, low-throughput and amplification bias. Fortunately, high-throughput sequencing (HTS)-based metagenomic analysis can overcome these problems and comprehensively provide information about the antibiotic resistomes in microorganisms from different samples^22^. For example, Ma et al. used a metagenomic approach and network analysis to establish a catalogue of antibiotic resistomes and perform host tracking in drinking water in 25 cities^23^. HTS-based metagenomic analysis has also been applied to activated sludge^4^, soil, and sediment samples^24^. Nevertheless, there are no reports on metagenomic analyses of RTE foods.

In this study, we investigated the distribution patterns of bacterial community and ARGs in 18 RTE foods (including RTE meat, RTE vegetable and RTE fruit) using the HTS-based metagenomic approaches. We then characterized the correlations between bacterial community and antibiotic resistome, and finally revealed the roles of bacterial community and MGEs in the resistome alteration in RTE foods. The data provide new insight into our standing of the distribution of bacterial community and ARGs and could be helpful to knowledge the complicated correlations among bacterial community, MGEs and ARGs in the RTE foods.

## Results

### Bacterial community structures in the RTE foods

**Figure 1:**
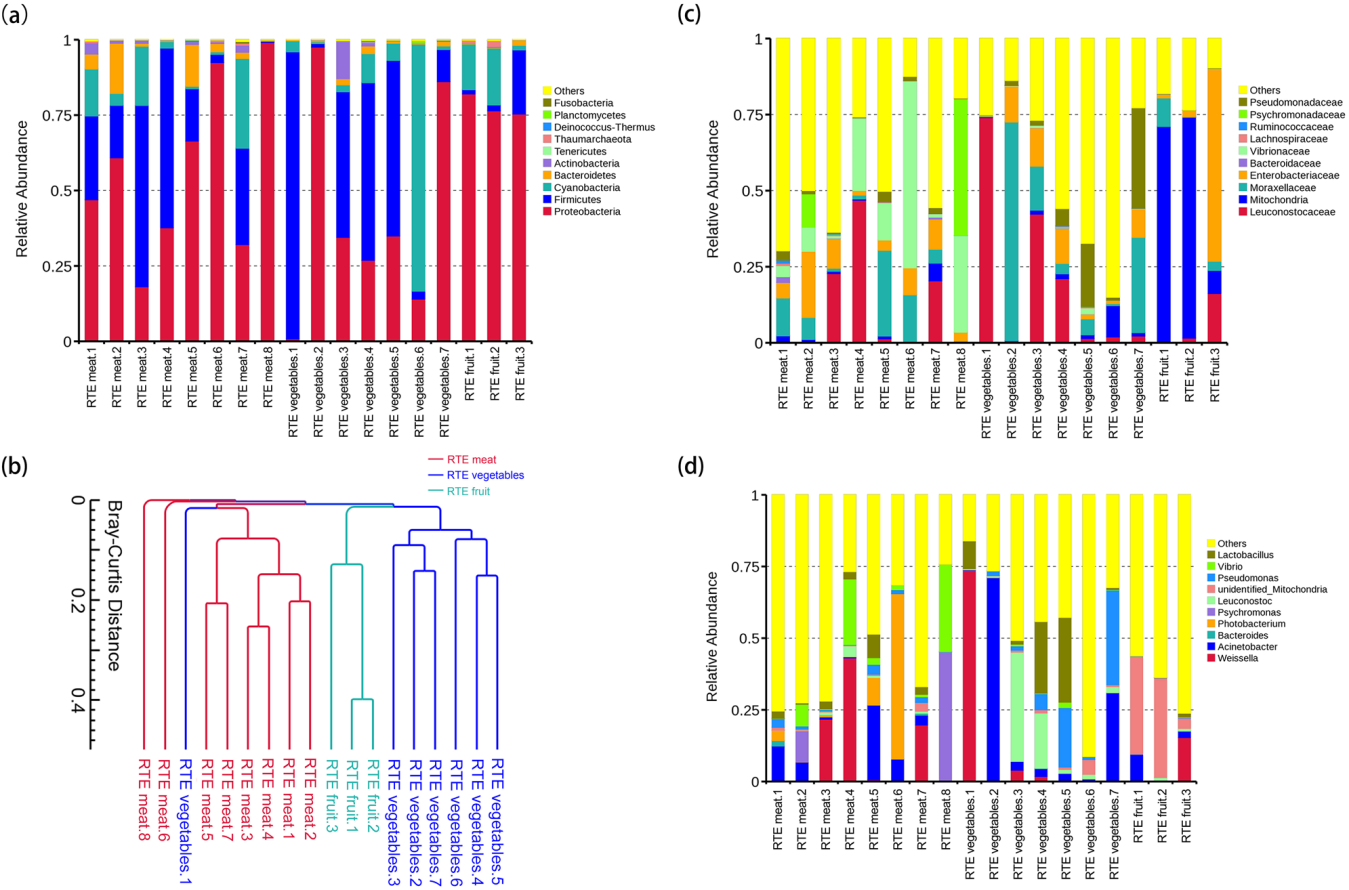
Distributions of bacterial community structures at different taxonomic levels (phylum, family and genus) in the 18 ready-to-eat samples. (**a**) The top 10 abundant bacterial community structures at the phylum level and the rest were set to “others”. (**b**) Sample classification using the unweighted Pair-group Method with Arithmetic Means based on the phyla level. (**c**) The top 10 abundant bacterial community structures at the family level and the rest were set to “others”. (**d**) The top 10 abundant bacterial community structures at the genus level and the rest were set to “others”.

First, 16S rRNA genes were extracted by sequence alignment against the SILVA_128_SSURef_Nr99 database. The results showed that the bacteria in RTE meat, vegetables and fruit were annotated to 41, 61 and 41 phyla, respectively (Supplementary Table S2). Figure 1a showed that the dominate phyla among RTE food samples was similar. The most abundant phyla in RTE foods were Proteobacteria (average = 54.6%), Firmicutes (average = 28.7%), Cyanobacteria (average = 11.9%), Bacteroidetes (average = 2.9%) and Actinobacteria (average = 1.4%) (Relative abundance ≥ 1%; Supplementary Table S2), reaching up to 90%. To study the similarity of different samples, a clustering tree of samples based on the phyla level was constructed using the unweighted Pair-group Method with Arithmetic Means (UPGMA), and the results showed that except for RTE vegetables.1, the bacterial community in the 17 samples could be then clustered into three groups: RTE meat, RTE vegetables and RTE fruit (Fig. 1b). These results were in good accordance with the sample types.

Figure 1c,d showed that the relative abundance of family and genus differed greatly among the RTE food samples. Vibrionaceae (average = 17.8%) and Leuconostocaceae (average = 11.5%) were the most abundant families in RTE meat samples, Leuconostocaceae (average = 20.5%) and Moraxellaceae (average = 18.2%) were the most abundant families in RTE vegetables samples, Mitochondria (average = 50.4%) and Enterobacteriaceae (average = 22.1%) were the most abundant families in RTE fruit samples (relative abundance ≥ 10%; Fig. 1c; Supplementary Table S3). *Weissella* (average = 10.7%) was the most abundant genus in RTE meat samples, *Acinetobacter* (average = 15.9%) and *Weissella* (average = 11.4%) were the most abundant genus in RTE vegetables samples, unidentified_*Mitochondria* (average = 24.1%) were the most abundant genus in RTE fruit samples (relative abundance ≥ 10%; Fig. 1d; Supplementary Table S4).

### Phylogenetic origins of antibiotic resistance genes in the RTE foods

**Figure 2:**
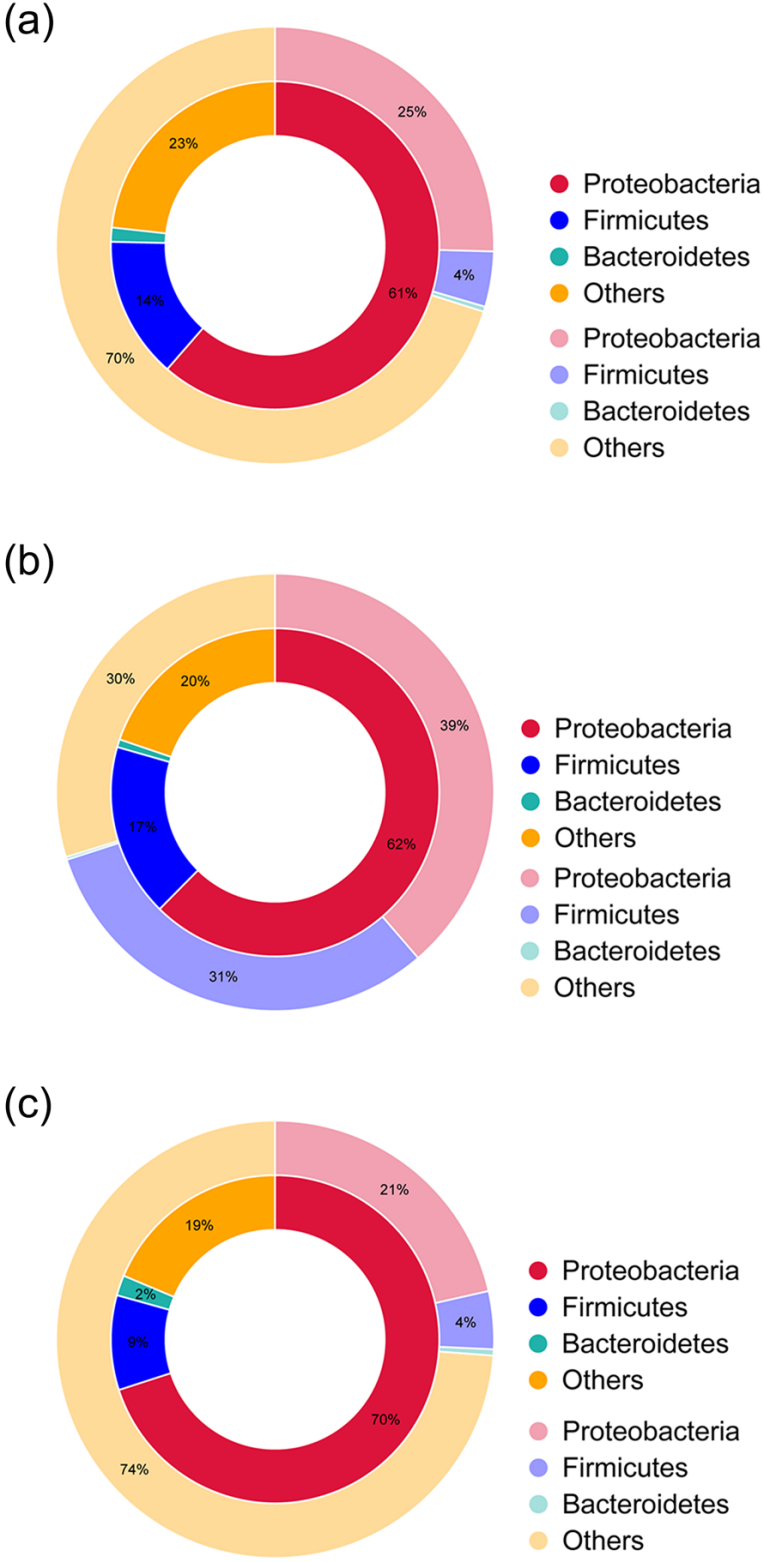
Comparison of the distribution of the antibiotic resistance genes (inner cycle) and the total microbial gene set (outer cycle) at the bacterial phylum level. (**a**) in ready-to-eat meat; (**b**) in ready-to-eat vegetables; (**c**) in ready-to-eat fruit. The ratios of genes (> 1%) assigned to each phylum are shown in the pie charts.

In order to compare the microbial origin of ARGs with the total microbial genes, the ARGs and the total microbial genes were assigned to different taxa with the assistance of the resistance gene identifier (RGI) and DIAMOND software. In RTE meat samples, the distribution of ARGs and total microbial genes at the phylum level was 61% and 25% for *Proteobacteria*, 14% and 4% for *Firmicutes*, respectively (Fig. 2a). In RTE vegetable samples, the assignment of ARGs and total microbial genes at the phylum level was 62% and 39% for *Proteobacteria*, 17% and 31% for *Firmicutes* (Fig. 2b). In RTE fruit samples, the distribution of ARGs and total microbial genes at the phylum level was 70% and 21% for *Proteobacteria* and 9% and 4% for *Firmicutes* (Fig. 2c). The results suggested that compared with the other genes, the ARGs were more prone to exist in *Proteobacteria* in RTE foods samples.

### Broad-spectrum profiles of ARG abundance in the RTE foods

**Figure 3:**
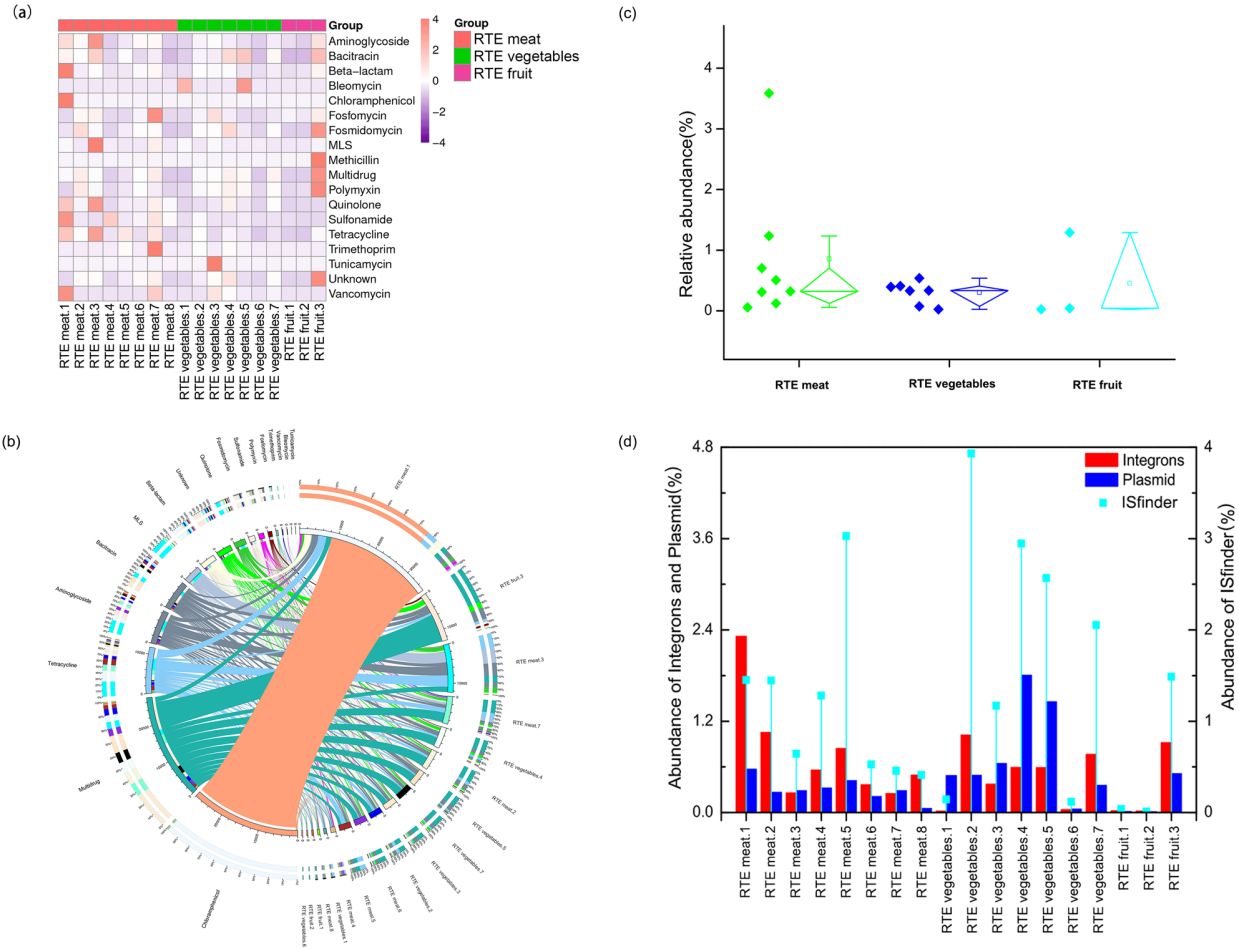
Characterization of antibiotic resistance gene (ARG) and mobile genetic elements (MGEs) in the 18 ready-to-eat (RTE) samples. (**a**) Broad-spectrum quantitative profile of ARG types (copy of ARG per copy of 16S rRNA gene) in the 18 samples. (**b**) Composition of ARG types in the 18 samples. (**c**) Comparison of the relative abundance of the antibiotic resistance genes in RTE meat, RTE vegetables and RTE fruit. Relative abundance refers to the proportion of the antibiotic resistance genes in the total genes in each type of RTE food samples. The line and the square in the diamond boxes denote the median and mean, respectively. The samples are shown as dots on the left. RTE meat: n = 8; RTE vegetables: n = 7; RTE fruit: n = 3. (**d**) The relative abundance of MGEs including integrons, plasmids and transposons in the 18 samples.

In total, 204 ARG subtypes belonging to 18 ARG types were detected in at least one of the 18 samples (Fig. 3a and Supplementary Table S5). The ARG diversity differed greatly among the 18 samples, and the number of ARG subtypes was in the range from 32 (RTE fruit.2) to 117 (RTE vegetables.5). The abundance of different ARG types in the samples varied greatly, from a ratio of 2.81 × 10^−5^ (Methicillin resistance genes in “RTE fruit.3”) to 7.7 × 10^−1^ (chloramphenicol resistance genes in “RTE meat.1”; Supplementary Table S6). Figure 3b showed the ARG-type composition in the 18 samples. In general, resistance genes associated with chloramphenicol, multidrug resistance, macrolide-lincosamide-streptogramin (MLS), aminoglycoside, bacitracin, tetracycline and β-lactam were more abundant and commonly distributed than other ARG types in these samples. Multidrug-ARGs, which encode resistance to multiple antimicrobial drugs, were found to be the most dominant in the RTE foods, with an abundance of 3.7 × 10^−2^–5.8 × 10^−1^ ratios (Supplementary Table S6). Additionally, the core-pan rarefaction curve indicated that as increase in the amount of sequencing data and sample size would not yield more new genes, illustrating that the current depth and the sample size was sufficient for microbial diversity investigation (Fig. 1S).

In order to compare the abundance of ARGs in the RTE meat, RTE vegetables and RTE fruit, we computed the relative enrichment of each of the genes based on sequencing coverage by using the original Illumina GA short reads. Figure 3c showed that the ARGs accounted for 0.86%, 0.30% and 0.45% of the total microbial genes in the RTE meat, RTE vegetables and RTE fruit, respectively. However, the RTE meat did not display the statistical difference with RTE vegetables and RTE fruit in AGRs abundance (Mann–Whitney test, *P* = 0.52445 and 0.35833, respectively). In addition, the relative abundance of MGEs differed greatly among the RTE food samples (Fig. 3d).

### Representative ARG types in the RTE foods

There were 204 ARG subtypes detected in the 18 samples, and we considered the top 10 most abundant ARG subtypes in each sample type to be representative ARGs (Table 1). For RTE meat, *catA1* was the most abundant ARG, but it was not among the top 10 abundant gene types in RTE vegetables or fruit (Table 1). The bacitracin resistance gene *bacA* was the second, first, and third most abundant ARG type in RTE meat, vegetables and fruit, respectively. The tetracycline resistance genes *tetM* (average = 2.22E−04), *tetL* (average = 2.20E−04) and *tetA* (average = 1.57E−04) were much more abundant in RTE meat than other types of RTE food. The MLS resistance gene *ermB* and the aminoglycoside resistance gene *aph(3`)-IIIa* were the fifth and seventh most abundant gene types in RTE meat, respectively. The multidrug resistance genes *acrB* and *emrD* were very common in all RTE foods (Supplementary Table S7), and both were in the top 10 most abundant gene types in RTE vegetables and fruit.

**Table 1:** Representative antibiotic resistance gene subtypes in different samples.

Sample types	ARG subtype^a^	ARG type	Abundance
Ready-to-eat meat	*catA1*	Chloramphenicol	0.003425681
	*bacA*	Bacitracin	0.00037352
	*tetM*	Tetracycline	0.000221939
	*tetL*	Tetracycline	0.000219554
	*ermB*	MLS	0.000218867
	*acrB*	Multidrug	0.000216345
	*aph3iiia*	Aminoglycoside	0.000189408
	*emrD*	Multidrug	0.000156569
	*tetA*	Tetracycline	0.000156563
	*lsa*	MLS	0.000137998
Ready-to-eat vegetables	*bacA*	Bacitracin	0.000467825
	*mexF*	Multidrug	0.000223517
	*acrB*	Multidrug	0.00019527
	*mexB*	Multidrug	0.000165355
	*mexW*	Multidrug	0.000141235
	*tet39*	Tetracycline	0.000123363
	*tolC*	Multidrug	0.000103659
	*ksgA*	Aminoglycoside	0.000100398
	*emrD*	Multidrug	8.25E−05
	*acrA*	Multidrug	7.55E−05
Ready-to-eat fruit	*emrD*	Multidrug	0.000502935
	*acrB*	Multidrug	0.000472052
	*bacA*	Bacitracin	0.000405067
	*tolC*	Multidrug	0.000353036
	*ksgA*	Aminoglycoside	0.000348471
	*acrA*	Multidrug	0.000312886
	*Bcr*	Bacitracin	0.000241684
	*macB*	MLS	0.000207836
	*mdfA*	Multidrug	0.000203576
	*mdtH*	Multidrug	0.000173293

^a^ARG, antibiotic resistance gene.

For RTE vegetables and fruit, representative ARG subtypes mainly belonged to multidrug resistance (*mexF*, *acrB, mexB*, *mexW*, *emrD*, *tolC*, *acrA*, *mdtH* and *mdfA*), bacitracin (*bacA*), tetracycline (*tet39*), aminoglycoside (*ksgA*) and macrolide (*macB*) resistance genes (Table 1). *acrB* and *bacA* were found in 100% of RTE food samples (Supplementary Table S7).

### Shared ARGs among RTE meat, vegetable and fruit samples

**Figure 4:**
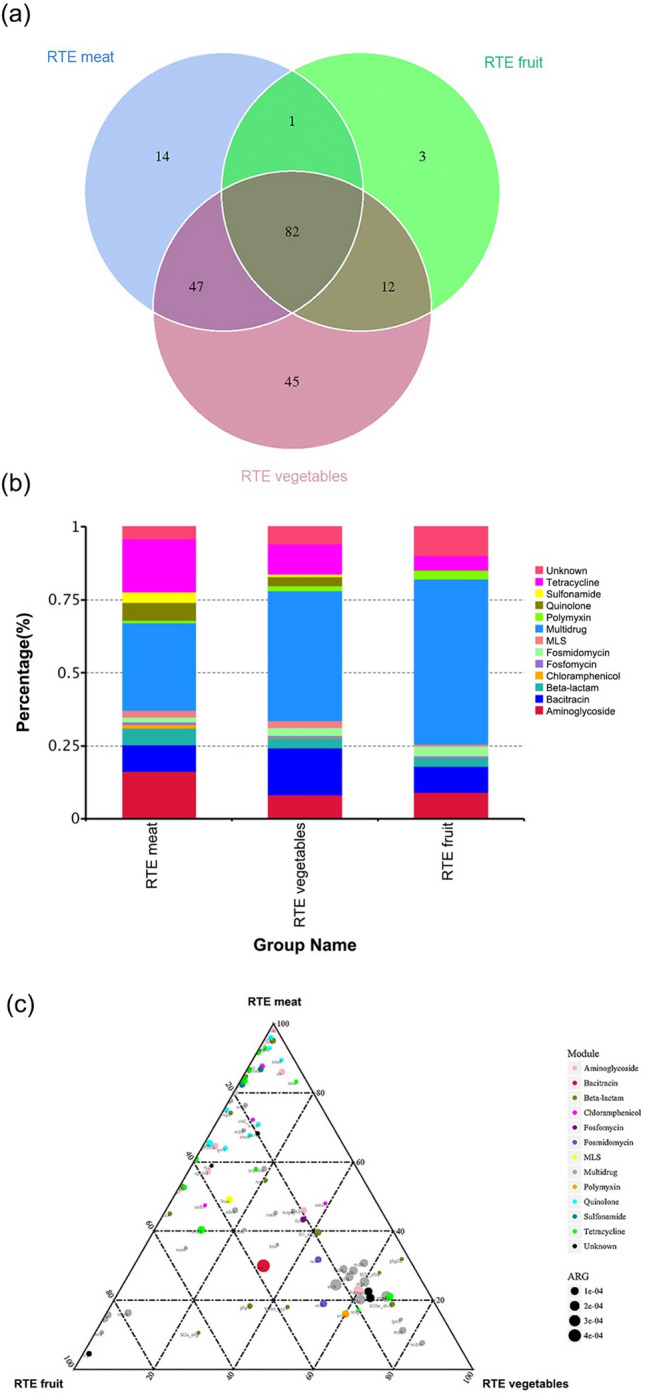
The composition and distribution of shared antibiotic resistance gene (ARG) in ready-to-eat (RTE) meat, vegetable and fruit samples. (**a**) Venn diagram showing the number of shared ARG subtypes among RTE meat, RTE vegetable and RTE fruit samples. (**b**) Composition of shared ARGs in RTE meat, RTE vegetable and RTE fruit. (**c**) Ternary plot comparing the abundance of the 82 shared ARG subtypes in RTE meat, RTE vegetable and RTE fruit. The sum of the abundance of one specific ARG in the three types of RTE foods was set as 100%. In the ternary plot, the percentage (%) of one specific ARG in each type of food was equal to its corresponding abundance divided by the sum of its abundance in the three types of RTE food. The symbol size indicates the abundance of ARGs (copies of ARG/copies of 16S rRNA gene).

Venn diagram was further applied to analyse the composition of shared ARGs among the RTE foods. We identified 82 AGRs belonging to 13 types shared by RTE meat, vegetables and fruit (Fig. 4a). The shared ARGs accounted for 56.94%, 44.09% and 83.67% of the total number of ARGs detected in RTE meat, vegetables and fruit, respectively (Supplementary Table S8). Among the shared ARGs, resistance genes related to multidrug resistance, bacitracin, aminoglycosides and tetracycline were the most abundant in RTE foods, accounting for > 60% of the total shared ARGs in each type of RTE food (Fig. 4b). Figure 4c shows a comparison of ARGs and their abundance among RTE meat, vegetable and fruit samples. The percentage of one specific ARG in each RTE food is equal to its corresponding abundance divided by the sum of its abundance in the three types of RTE food. The results showed that the abundance of genes related to tetracycline (average = 1.8E−1), sulphonamides (average = 3.7E−2), beta-lactams (average = 5.6E−2), aminoglycosides (average = 1.6E−1) and quinolone (average = 0.18) was much higher in RTE meat than in RTE vegetables or fruit. However, the abundance of multidrug resistance genes (average = 5.7E−1) was much higher in RTE vegetables and fruit than in RTE meat.

### Co-occurrence between ARG subtypes and microbial taxa in the RTE foods

**Figure 5:**
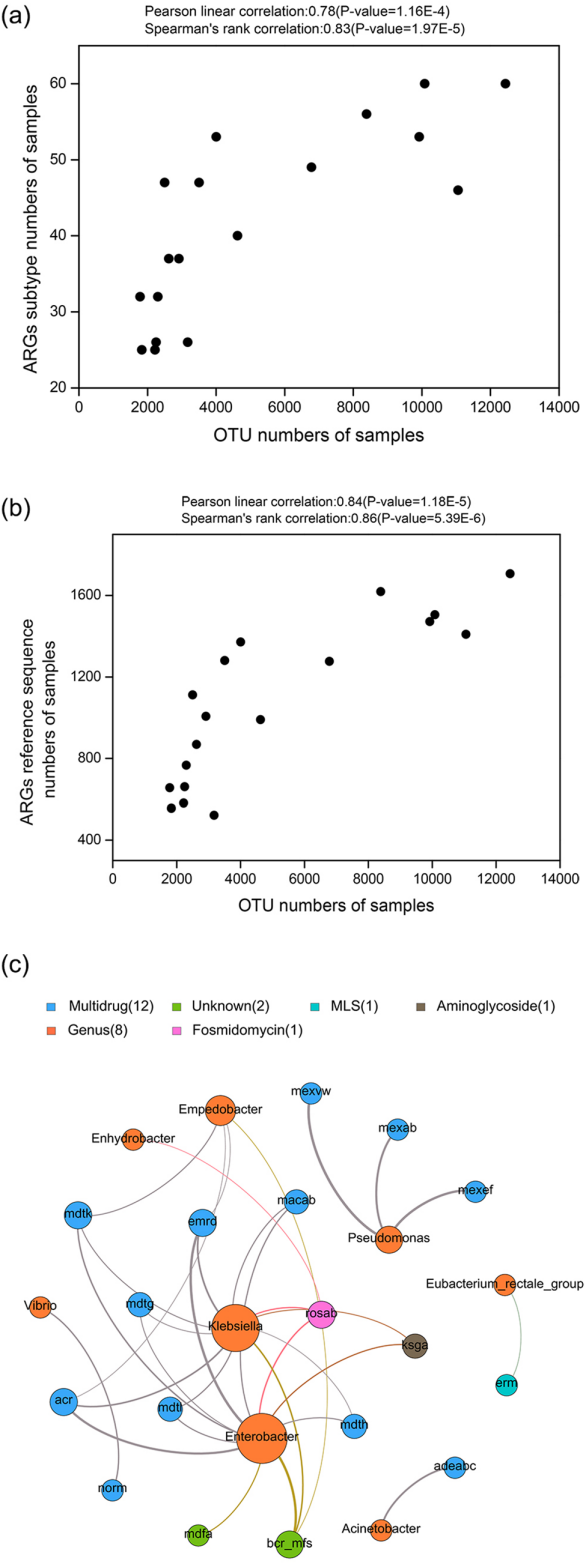
The correlation analysis and network analysis between antibiotic resistance genes (ARGs) and microbial taxa in ready-to-eat (RTE) foods. (**a**) Correlation analysis of the ARGs diversity with microbial diversity at subtype level. (**b**) Correlation analysis of the ARGs diversity with microbial diversity at reference sequence level. (**c**) Network analysis showing the co-occurrence patterns between antibiotic resistance gene subtypes and microbial taxa in RTE foods. The nodes were colored according to antibiotic resistance gene types and genus. A connection represents a strong (Spearman’s correlation coefficient ρ > 0.8) and significant (*P* value < 0.01) correlation. The size of each node is proportional to the number of connections.

The diversity of microbial community and ARGs in representative RTE food were displayed in Supplementary Table S9. There was a significant Spearman’s rank correlation (Spearman’s ρ = 0.83 ~ 0.86, *P* value < 1.0E−5) between the microbial diversity and the ARGs diversity (Fig. 5a,b). The network analysis approach was applied to explore the co-occurrence patterns between ARG subtypes and microbial taxa (Fig. 5c). According to a previous study^23,24^, it was hypothesized that the non-random co-occurrence patterns between ARGs and microbial taxa could indicate possible host information for AGRs if the ARGs and co-existing microbial taxa possessed significantly similar abundance trends among the different environments (Spearman’s ρ > 0.8, *P* value < 0.01). Forsberg verified that some specific microbial taxa carrying some specific ARGs led to the corresponding similar abundance trends^25^. The modularity index of 0.359 indicates that the co-occurrence pattern network has a modular structure^26,27^. The detailed co-occurrences between ARG subtypes and microbial taxa are summarized in Supplementary Table S10. A total of 8 genera were identified as possible host ARGs based on the co-occurrence results, and *Vibrio*, *Enhydrobacter*, *Acinetobacter*, *Klebsiella*, *Pseudomonas* and *Enterobacter* were present in all samples. *Klebsiella* was the host of multidrug resistance genes (*Acr*, *macAB*, *emrD*, *mdtG*, *mdtH*, *mdtL*, *mdtK*), the fosmidomycin resistant gene *rosab*, the aminoglycoside resistance gene *ksgA* and other resistance gene *bcr_mfs*. *Enterobacter* appeared to possess diverse resistance genes, including multidrug resistance genes (*mdtK*, *emrD*, *mdtG*, *mdtL*, *mdtH*, *macAB*, *mdfA* and *Acr*), the fosmidomycin resistance gene *rosab*, the aminoglycoside resistance gene *ksgA* and other resistance genes (*bcr_mfs*). By contrast, *Pseudomonas* mainly possessed multidrug resistance genes (*mexEF*, *mexVW* and *mexAB*), whereas the multidrug resistance gene (*norM*) was only associated with *Vibrio*. *Acinetobacter* was found to be the host of multidrug resistance gene *ADeABC*. *Eubacterium rectal* group appeared to carry the MLS resistance gene *erm* while *Enhydrobacter* was found to be the host of the fosmidomycin resistance gene *rosab*. *Empedobacter* harboured the multidrug resistance genes (*Acr*, *mdtK* and *emrD*) and other resistant gene *bcr_mfs*.

### Correlations among bacterial community, MGEs and ARGs in the RTE foods

**Figure 6:**
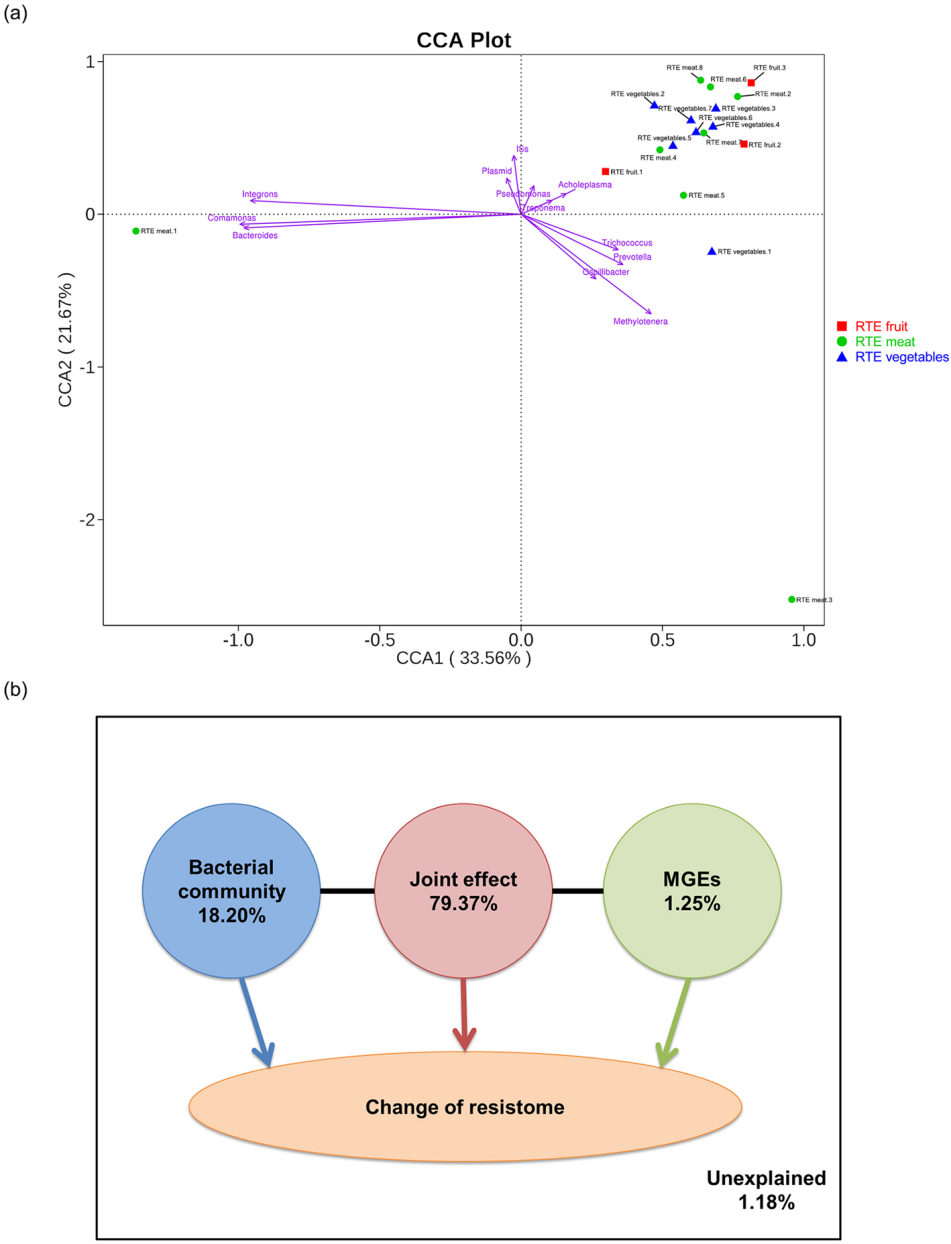
The correlation analysis among bacterial community, mobile genetic elements (MGEs) and antibiotic resistance genes in the ready-to-eat (RTE) foods. (**a**) Canonical correspondence analysis (CCA) illustrating relationships between the genera and MGEs in RTE foods. (**b**) Variation partitioning analysis differentiating effects of bacterial community and MGEs on the resistome alteration.

Canonical correspondence analysis (CCA) and variation partitioning analysis (VPA) were performed to further analyze the potential links among bacterial community structure, MGEs and ARGs distribution. CCA showed that *Methylotenera*, *Trichococcus*, *Prevotella*, *Osillibacter*, *Integrons*, *Comamonas*, *Bacteroides*, transposons (ISs), plasmid, *Acholeplasmia*, *Pseudomonas* and *Treponema* played important roles in shaping the ARG profiles in the RTE food samples, which contributed 98.82% of ARG shifts (Fig. 6a,b). *Methylotenera*, *Trichococcus*, *Prevotella* and *Osillibacter* positively correlated with RTE vegetables.1 and RTE meat.3, which indicated that their importance in determining ARG compositions in these two samples. Integrons, *Comamonas*, *Bacteroides*, transposons and plasmid positively correlated with RTE meat.1. *Acholeplasma*, *Pseudomonas* and *Treponema* were positively correlated with the remaining 15 samples. VPA further revealed that the joint effects of bacterial community shift and MGEs change contributed more (79.37%) to the resistome alteration than bacterial community shift (18.2%) and MGEs change (1.25%; Fig. 6b).

## Discussion

The food chain is considered to be an important contributor to the development and dissemination of antibiotic resistant microbes^28,29^. There have been many reports on microbial community in raw and RTE foods, but most were based on traditional culture or PCR methods^20,30,31^. In recent years, although metagenomic sequencing technology has been applied to investigate microbial community in food, most studies have focused on tracking changes in complex microbial community in fermented foods^32,33^. Moreover, RTE foods have not yet been thoroughly analysed, which are directly eaten without further treatment. The present study showed that the most abundant phyla composition in RTE foods were similar, which were Proteobacteria, Firmicutes, Cyanobacteria, Bacteroidetes and Actinobacteria. By comparison, many previous studies have reported the dominant phyla was Proteobacteria in commercial salad leaf vegetables^34^, RTE salad^35^, fresh spinach stored at low temperature^35^, and RTE lettuce^36^. However, the bacterial community at the family and genus levels varied differently among the RTE food samples. This indicated that the lower the classification level of microbial community structure in RTE food, the higher the richness of microbial groups was observed. The differences might be due to differences in the food types, growing conditions of food, food processing, or preservation conditions^37^. In contrast, the microbial community in RTE food at family and genus level was previously less studied. HTS-based metagenomic approach can comprehensively reflect microbial community structure. Additionally, although the 18 samples in this study were collected from six different supermarkets, most of the samples (17/18) were well clustered according to the sample type. The reason why RTE vegetables.1 was clustered with RTE. meat samples may be due to the deviation caused by the food processing. For example, the staffs in the supermarket that have treated the meat have also processed RTE vegetables.1.

To our knowledge, this is also the first comprehensive analysis of the bacterial composition in the RTE foods by the metagenomic approach. 16S rDNA sequencing is another widely used method for identifying food microbial communities. The cost is relatively lower and bioinformatic tools designed for sequencing data analysis are free and easy to operate. Nevertheless, the selection of the hypervariable region for 16S rDNA has been more dependent on published or in-house designed protocols^38^.By contrast; the metagenomic approach can provide the comprehensive information on genes, structure and organization of the genomes, microbial community structure and evolutionary relationships present in the sample^17^.

In this study, the top 10 most abundant ARG subtypes in each sample type were considered to be representative ARGs (Table 1). The relative abundances of the 10 ARG subtypes ranged from 7.55 × 10^–5^ to 3.42 × 10^–3^ copy of ARG/ copy of 16S rRNA gene. Typically, the relative abundance of ARGs in unpolluted environments ranges from 10^–8^ to 10^–6^ copies/16S rRNA, whereas concentrations in highly contaminated sites are often several orders of magnitude higher (e.g., 10^–4^)^39^. Previous study revealed that the relative abundance of ARGs (*sulI*, *sulII*, *blaTEM-1*,*tetA*, *tetO*, *tetQ*, and *tetW*) ranged from 10^–6^ to 10^–1^ gene copies per 16S rRNA in the four major Chinese carp, while the relative abundance of ARGs (*qac*, *sul*, *erm* and *tetM*) ranged from 1.46 × 10^–7^ to 4.6 × 10^1^ copies/ 16S rRNA in raw and fresh foods^16,40^. Yao et al. also reported that the relative abundances of *qnrD*, *qnrS*, *mexF*, *ermA*, *ermB*, *mefA*, *sul1*, and *sul2* in most landfills were > 10^–4^ copies per 16S rRNA, suggesting the presence of highly contaminated ARGs^39^. Therefore, we should pay more attention on the ARGs with relative abundance > 10^–4^ copies per 16S rRNA. However, according to previous reports based on PCR methods, well-studied ARG types mainly include resistance genes related to tetracycline, sulphonamides, β-lactams, macrolides, methicillin and aminoglycosides (Supplementary Table S11). Tetracycline and macrolide resistance genes account for nearly 80% of previous studies (Supplementary Table S11). Meanwhile, no more than 50 ARG subtypes are listed in previous studies (Supplementary Table S11). Due to the limited availability of primers used in traditional PCR-based methods, a mere snapshot of possible ARG profiles has been determined for RTE foods, such as *tetM*, *ermB* and *aac(6′)Ie‐aph(2″)* in RTE dishes^41^. However, HTS-based metagenomic analysis could capture a more comprehensive picture of the correlations among ARG profiles without PCR bias, and can simultaneously analyse 18 ARG types, consisting of 204 ARG subtypes. Furthermore, the results of metagenome would provide the new research perspective in the future. For example, our study revealed that the multidrug resistance genes *acrB* (relative abundance = 1.95 × 10^–4^, 4.72 × 10^–4^, respectively) and *ksgA* (relative abundance = 1 × 10^–4^, 3.48 × 10^–4^, respectively) were both among the top 10 most abundant gene types in RTE vegetables and fruit, which has been barely reported based on PCR-based approaches and could be researched deeply in future.

Multidrug-ARGs were found to be the most dominant in RTE foods with an abundance of 3.7 × 10^−2^–5.8 × 10^−1^ ratios. By contrast, environmental data showed that the dominant ARG types in drinking water, animal faeces and activated sludge were multidrug-ARGs, tetracycline-ARGs and aminoglycoside-ARGs, respectively^23,24^. Many previous studies have reported the prevalence of multidrug-ARGs in RTE foods4^42–46^. Multidrug resistance has been detected in RTE turkey meat products, and multidrug resistance was higher than in raw meat^44^. Diverse multidrug resistant strains were isolated from RTE Paan, including *Salmonella Teko* and *Salmonella Virchow*^38^. Moreover, fishery products such as RTE raw fish and shrimp were once considered to be a vehicle for transmission of multidrug resistant pathogens^42,43^. Carvalheira et al. reported that lettuce and fruits were the source of multidrug resistant *Acinetobacter spp.* and 29.8% of the strains were classified as multidrug-resistant^47^. Due to the long-term use of antibiotics in human and veterinary medicine, the problem of microbial resistance has become increasingly significant. Many studies have demonstrated that antibiotic resistance genes could spread by horizontal gene transfer between different bacterial communities, leading to the widespread prevalence of drug resistance genes and the emergence of multidrug resistance^48,49^. The multidrug-resistant strains in the foods may be a threat to public health, which may transmit these pathogens to human beings and to the environments that surround them^44,45^. The multidrug resistance gens identified in the present research may be intrinsic to bacteria associated with the food itself or result from environmental contamination or transmission by food handlers. However, further systematic studies are needed to track contamination pathways of AR bacteria and ARGs in food production and to determine whether these resistance genes can cause potential harm to humans.

By studying the components in the shared ARGs, the results showed that resistance genes related to multidrug resistance, bacitracin, aminoglycosides and tetracycline were most abundant in RTE foods. The shared ARGs might be caused by bacterial community shift or MGEs alteration among food, the environment, and human manipulation^50^. Tetracycline, sulphonamides, beta-lactams, aminoglycosides and quinolone were found to be much higher in RTE meat than in RTE vegetables or fruit, which may be caused by the overuse and misuse of antibiotics in the livestock breeding^51^.

We utilized a correlation-based network approach to explore the ARG-species co-occurrence patterns in RTE foods, which helped to propose a useful reference for future studies on risk management of ARGs in RTE foods. Based on the network analysis data obtained in this study, some of the identified ARG hosts have been reported in previous studies. For instance, the resistance gene *MexVW* is commonly carried by *Pseudomonas*^52^, and the resistance gene *emrD* has been reported in *Enterobacter*^53^. Some hosts have been rarely reported previously and should receive more attention in further studies, such as *Empedobacter*. The dominant genera, *Acinetobacter* (average = 9.0%), *Pseudomonas* (average = 3.6%) and *Vibrio* (average = 2.9%) were found to carry multidrug resistance genes (*MexVW*, *MexEF*, *AdeABC* and so on), which have been always investigated in the RTE foods^54–56^ (Supplementary Tables S2 and S10). Some *Acinetobacter spp.* (e.g., *A. baumannii*), *Pseudomonas spp.* (e.g., *P. aeruginosa*) and *Vibrio spp.* (e.g., *V. parahaemolyticus*) have been considered as opportunistic pathogens, and their capacity of thriving in RTE foods could increase the risks of the exposure and spread of ARGs between the RTE foods and human beings. Thus, we should focus on theses ARG-carrying dominant bacteria observed in RTE foods and how to develop the effective strategies to control AR.

The results from the VPA analysis showed that the joint effects of bacterial community shift and MGEs mainly explain the resistome alteration in the RTE food samples. It is known that MGEs alteration were found to affect the ARG profiles directly in commensal and/or potential pathogens found in the food, such as chicken and vegetable^57,58^. However, the role of the bacterial community shift for ARGs change in the food was few explored. By contrast, many previous study revealed that the bacterial community played an important role in the shaping the profiles of ARGs among different microorganisms in the environments. Han et al. reported that community composition was the main factor driving changes in ARG abundance in a mariculture sediment^59^. Liao et al. found that shifts in bacterial community composition were associated with the maintenance of ARGs in food waste composting^60^. Thus, further work is needed to better understand how bacterial community shift drive ARG alteration in the food samples. Additionally, the CCA analysis explained that *Acholeplasma*, *Pseudomonas* and *Treponema* were positively correlated with the most of the studied RTE food samples. *Pseudomonas* was the dominant genus in RTE food samples, harboring the multidrug resistant genes, which was presented in all RTE samples (Supplementary Table S10). This may illustrate the phenomena that multidrug-ARGs were the most abundant in RTE foods. Similarity, it was also reported that *Pseudomonas* carried the multidrug resistant genes^52,61^.

This is the first study of the distribution of the microbial community and ARGs between RTE foods using the metagenomic approach. These findings could provide a broader perspective on the ARGs and AR bacteria in RTE foods for the government regulation and risk assessment. Future study with larger sample size would help to validate the findings of the current study and reveal more information and biological significance.

## Methods

### Sampling information and DNA extraction

Basic information on the 18 RTE food samples included in this study is summarized in Supplementary Table S1, including 8 RTE meat (roast pork, roast duck, roast chicken and salmon sushi), 7 RTE vegetables (cucumber, kelp, lotus root and so on) and 3 RTE fruit (fresh-cut hami melon). These samples were randomly purchased from 6 local supermarkets in southern China. The samples were placed in separate sterile plastic bags to prevent cross-contamination and immediately transported to the laboratory in a cooler with ice packs and processed within two hours. Each sample unit was at least 500 g. Each sample was placed in a sterile culture dish and then wiped off the food surface with a disposable sterile specimen collection swab (Copan, Italy) dipped in saline. The wiped sterile specimen collection swabs were placed in sterile 50 ml centrifuge tubes. All these samples were stored at -80 °C, and then transported to Novogene (Beijing, China) with dry ice.

Genomic DNA was extracted using the cetyltriethylammnonium bromide standardization operation protocol (Supplementary Text S1) at Novogene (Beijing, China). DNA degradation degree and potential contamination was monitored on 1% agarose gels. Meanwhile, DNA concentration was measured using Qubit dsDNA Assay Kit in Qubit 2.0 Flurometer (Life Technologies, CA, USA). OD value is between 1.8 and  2.0, DNA contents above 1 μg are used to construct library.

### Metagenomic sequencing and bioinformatics analysis

Metagenomic sequencing was performed on an Illumina Novaseq platform (paired-end 150 bp reads) by Novogene (Beijing, China). The generated raw sequences containing 10 or more unknown nucleotides (`N`) or low-quality bases (default quality threshold value ≤ 38) or contaminated by adapters (15-bp overlap) were removed for each sample (FASTQ format) using the quality control pipeline recommended by the sequencing institution. Considering the possibility of host pollution in samples, clean data needed to be blasted against the host database with default parameters using Bowtie 2.2.4 software to filter the reads that were of host origin. The parameters were as follows: –end-to-end, –sensitive, -I 200, -X 400^62,63^. In addition, trimmed metagenome reads were assembled into scaffolds using SOAPdenovo software (B2.04)^64^, and the assembled scaffolds from N connections were then interrupted, leaving scaftigs without N^65–67^. All scaftigs were subjected to prediction of open reading frames (ORFs) using MetaGeneMark (V2.10) software^65,66,68,69^. The complete data totalled 270 Gb, which is the largest sequence dataset reported to date for a study on ARGs in RTE food samples.

The SILVA_128_SSURef_Nr99 database was used to characterize the structure of the bacterial community, where the reads was blasted using the BLAST software (V2.2.25) and the parameters were as follows: -p blastn -m 8 -a 4 -e 1e-20 -b 50, identity ≥ 90, alignment ≥ 75^70,71^. Briefly, 16S rRNA gene-like sequences from the BLAST results were assigned to NCBI taxonomies, as implemented in the R “Vegan” package, using the unweighted Pair-group Method with Arithmetic Means (UPGMA) algorithm^72^. All metagenomic sequencing data were searched for ARGs against the Comprehensive Antibiotic Resistance Database (CARD) using the RGI software with an E-value ≤ 1e−30^71,73^. A package of customized scripts was developed for automatic classification of identified ARG-like sequences into 18 “ARG types” (e.g., tetracycline resistance gene) and 204 “ARG subtypes” (e.g., *tetA*, *tetB*, etc.)^23,24^. The sequence identity was ≥ 90%, and the alignment length was ≥ 25 amino acids^24^. To assess the ARG distribution in all samples, the abundance of ARGs was expressed as “copy of ARG/copy of 16S rRNA gene” and calculated using the following equation:

$$\mathrm{Abundance}=\sum_{1}^{\mathrm{n}}\frac{{N}_{\mathrm{ARG}-\mathrm{like sequence}}\times {L}_{\mathrm{reads}}/{L}_{\mathrm{ARG reference sequence}}}{{N}_{16\mathrm{S sequence}}\times {L}_{\mathrm{reads}}/{L}_{16\mathrm{S sequence}}}$$

where *N*_ARG-like_ sequence is the number of the ARG-like sequence annotated as one specific ARG reference sequence; *L*_ARG reference sequence_ is the sequence length of the corresponding specific ARG reference sequence; *N*_16S sequence_ is the number of the 16S sequence identified from the metagenomic data; *L*_16S sequence_ is the average length of 16S rRNA genes (1432 bp) in Greengenes database; *n* is the number of the mapped ARG reference sequence belonging to the ARG type or subtype and *L*_reads_ is the sequence length of the Illumina reads used in the present study^24^.

To determine the diversity and abundance of MGEs, BLAST tool was applied to annotate sequencing reads against offline databases of MGEs (including integrons, plasmids and transposons), and the parameters were as follows: –identity 90 –alignlen 754^4,24,71^. The referenced MGEs databases were NCBI NR (ftp://ftp.ncbi.nlm.nih.gov/blast/db/nr.gz), NCBI plasmids (ftp://ftp.ncbi.nih.gov/genomes/Plasmids/), and Isfinder (https://www-is.biotoul.fr/), respectively.

### Statistical and network analyses

In order to calculate the abundance of genes, the ORF prediction results assembled by each sample were de-redundant using the CD-HIT software to obtain the non-redundant initial gene catalogue. By default, clustering was performed with indentify 95% and coverage 90%, and the longest sequence was selected as the representative sequence. The Bowtie2 was used to compare the clean data of each sample to the initial gene catalogue, and calculate the number of reads for the gene comparison in each sample. Filter out the genes that support the number of reads ≤ 2 in each sample to obtain the gene catalogue (unigenes) for subsequent analysis. The abundance of each gene in each sample was calculated based on the number of reads and the length of genes on the alignment. The calculation equation was shown as follows:

$${\mathrm{G}}_{\mathrm{k}}=\frac{{r}_{k}}{{L}_{k}}\cdot \frac{1}{\sum_{i=1}^{n}\frac{{r}_{i}}{{L}_{i}}}$$

where *r* is the number of reads annotated to genes, *L* is the length of genes.

Then, the DIAMOND software was used to compare the unigenes with the MicroNR database to obtain the species annotation. The parameters were as follows: blastp, evalue ≤ 1e−5. For the alignment results of each unigenes, the alignment results of evalue  ≤ min evalue × 10 were selected for the further analysis. After filtering, as each unigenes may have many comparison results and obtained different information of species classification. In order to ensure its biological significance, LCA algorithm was applied to take the first branch of classification level as the species annotation information of the unigenes. The abundance of the species was obtained by combing the abundance of the unigenes. In order to analysis the taxonomic affiliation of ARGs, the unigenes was firstly blasted against the CARD using the RGI software to search for ARGs, and the parameters were as follows: evalue ≤ 1e−30^74^. According to the comparison result of RGI, combined with the abundance of unigenes that annotated to antibiotic resistance ontology (ARO), the relative abundance of each ARO was calculated to obtain the abundance of ARGs. The species annotation information of ARO was obtained from the unigenes annotated ARO. Finally, we used the script software to draw the picture of svg at phylum level.

Ternary plots were implemented in the R “ggtern” package based on the abundance matrix of shared ARG subtypes in all RTE food samples^24^.

CCA was performed to determine the potential links among bacterial community structure, MGEs and ARGs distribution. VPA was further conducted to determine relative contribution of bacterial community shift and MGEs changes to the resistome alteration. CCA and VPA were conducted using the R “vegan” package^75,76^.

A co-occurrence network was employed to visualize the correlation between ARG subtypes and microbial taxa. A connection indicated a strong (ρ > 0.8) and significant (*P* value < 0.01) Spearman’s correlation. The size of each node was proportional to the number of connections, and the thickness of each connection (i.e., edge) between two nodes was proportional to the absolute value of the Spearman’s correlation coefficient. Co-occurrence networks were visualized using the Gephi interactive platform^77,78^. Various parameters (e.g., number of nodes and edges, average path length, clustering and modularity coefficient) were calculated using the R “igraph” package^79^.

## Supplementary information

Supplementary Information.

## Data Availability

All sequencing data has been deposited in the European Nucleotide Archive (ENA) under accession code PRJEB33440.
