# Supplementary material for: Metagenomic characterization of bacterial community and antibiotic resistance genes in representative ready-to-eat food in southern China
Source: Sci Rep. 2020 Oct 22;10:15175. doi: 10.1038/s41598-020-72620-4 (PMC7581714; doi:10.1038/s41598-020-72620-4)
Supplement: Supplementary file 1 — Supplementary Information. [file 41598_2020_72620_MOESM1_ESM.docx]

**Metagenomic characterization of bacterial community and antibiotic resistance genes in representative** **ready-to-eat food in southern China**

YiMing Li^1+^, WeiWei Cao^2+^, ShuLi Liang^3^, Shinji Yamasaki^4^, Xun Chen^2^, Lei Shi^2*^ and Lei Ye^2*^

*Corresponding author

**Text S1** Cetyltriethylammnonium bromide (CTAB) standardization operation protocol

1) Absorb 1000 μl CTAB lysate into 2.0 ml EP tube, add lysozyme. When the appropriate sample is added into the lysate, take a 65℃-water bath and mix it up and down several times during the process, so that the sample can be fully cracked.

2) Centrifugal supernatant, add phenol (pH 8.0) chloroform: isoamyl alcohol (25:24:1), reversed and mixed, 12000×rpm centrifuge for 10 min.

3) Remove the supernatant and add chloroform: isoprene (24:1), mix them up and down, then centrifuge at 12000×rpm for 10min.

4) Extract the supernatant to 1.5 ml centrifuge tube, add isopropanol, and shake up and down, precipitation in -20℃.

5) Centrifuge at 12000×rpm for 10 minutes, and pour out the liquid. Be careful not to pour out the precipitate. Wash with 1 ml 75% ethanol for two times, a small amount of remaining liquid can be collected by centrifugation again, and then sucked out with the tip of the gun.

6) Blow-dry the super-clean bench or air dry at room temperature (the DNA sample should not be too dry, otherwise it will be difficult to dissolve)

7) DdH2O dissolved DNA samples were added and incubated for 10 min at the level of 55-60℃.

8) Add RNase A 1μl to digest RNA, and leave at 37℃ for 15 minutes.

**
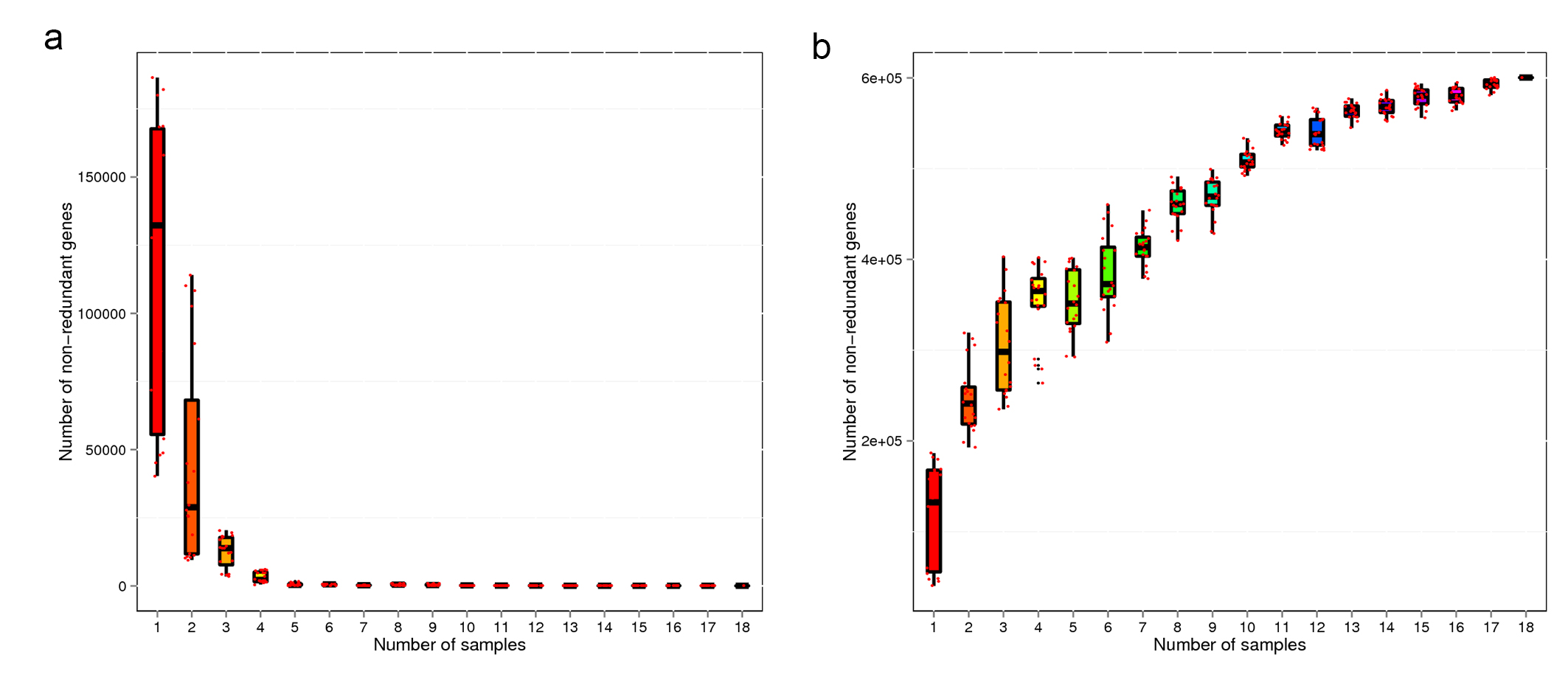
**

**Figure 1S.** The core-pan rarefaction curve showing the sequencing depth is sufficient to investigate the microbial diversity. a. core rarefaction curve; b. pan rarefaction curve.

**Table. S1.** Basic information summary of these ready-to-eat food samples

| Sample ID | Sample type | Sampling time | Clean Dataset size (Gb) | Sequencing technology | Remark |
| --- | --- | --- | --- | --- | --- |
| ^a^RTE meat.1 | Ready-to-eat meat | July, 2017 | 16 | Illumina, NovaSeq PE150 | packaged roast pork from supermarket A, Tianhe District, Guangzhou, China |
| RTE meat.2 |  | January, 2018 | 13 |  | packaged roast pork from supermarket B, Huangpu District, Guangzhou, China |
| RTE meat.3 |  | January, 2018 | 13 |  | packaged roast pork from supermarket C, Tianhe District, Guangzhou, China |
| RTE meat.4 |  | December, 2017 | 13 |  | packaged roast pork from supermarket D, Tianhe District, Guangzhou, China |
| RTE meat.5 |  | January, 2018 | 10 |  | packaged roast pork from supermarket E, Tianhe District, Guangzhou, China |
| RTE meat.6 |  | July, 2017 | 10 |  | packaged salmon sushi from supermarket C, Tianhe District, Guangzhou, China |
| RTE meat.7 |  | July, 2017 | 19 |  | packaged roast duck from supermarket C, Tianhe District, Guangzhou, China |
| RTE meat.8 |  | January, 2018 | 11 |  | packaged roast chicken from supermarket B, Huangpu District, Guangzhou, China |
| RTE vegetables.1 | Ready-to-eat vegetables | September, 2017 | 7 |  | packaged Chinese salad (cucumber; lotus root; kelp) from supermarket A, Tianhe District, Guangzhou, China |
| RTE vegetables.2 |  | September, 2017 | 10 |  | packaged Chinese salad (cucumber; lotus root) from supermarket B, Huangpu District, Guangzhou, China |
| RTE vegetables.3 |  | September, 2017 | 12 |  | packaged Chinese salad (cucumber; lotus root; kelp; white radish; carrot; garlic) from supermarket C, Tianhe District, Guangzhou, China |
| RTE vegetables.4 |  | January, 2018 | 9 |  | packaged Chinese salad (lotus root) from supermarket B, Huangpu District, Guangzhou, China |
| RTE vegetables.5 |  | January, 2018 | 9 |  | packaged Chinese salad (kelp; carrot; garlic) from supermarket C, Tianhe District, Guangzhou, China |
| RTE vegetables.6 |  | January, 2018 | 7 |  | packaged Chinese salad (kelp) from supermarket D, Tianhe District, Guangzhou, China |
| RTE vegetables.7 |  | January, 2018 | 13 |  | packaged Chinese salad (cucumber; lotus root) supermarket E, Tianhe District, Guangzhou, China |
| RTE fruit.1 | Ready-to-eat fruit | August, 2017 | 35 |  | packaged fresh-cut hami melon from supermarket A, Tianhe District, Guangzhou, China |
| RTE fruit.2 |  | August, 2017 | 45 |  | packaged fresh-cut hami melon from supermarket F, Huangpu District, Guangzhou, China |
| RTE fruit.3 |  | August, 2017 | 18 |  | packaged fresh-cut hami melon from supermarket C, Tianhe District, Guangzhou, China |

^a^RTE, ready-to-eat

**Table. S2.** Relative abundance of bacterial community at the phylum level in these ready-to-eat food samples

| Taxonomy | ^a^RTE meat.1 | RTE meat.2 | RTE meat.3 | RTE meat.4 | RTE meat.5 | RTE meat.6 | RTE meat.7 | RTE meat.8 | RTE vegetables.1 | RTE vegetables.2 | RTE vegetables.3 | RTE vegetables.4 | RTE vegetables.5 | RTE vegetables.6 | RTE vegetables.7 | RTE fruit.1 | RTE fruit.2 | RTE fruit.3 |
| --- | --- | --- | --- | --- | --- | --- | --- | --- | --- | --- | --- | --- | --- | --- | --- | --- | --- | --- |
| Proteobacteria | 0.469487184 | 0.608478448 | 0.181028219 | 0.376522568 | 0.66392938 | 0.924287979 | 0.320798996 | 0.990624668 | 0.008041295 | 0.975557202 | 0.344703942 | 0.268771902 | 0.349399598 | 0.140146984 | 0.860849607 | 0.820158342 | 0.764067793 | 0.753669198 |
| Firmicutes | 0.277744613 | 0.174006942 | 0.601314266 | 0.595664039 | 0.17359709 | 0.027217683 | 0.319584675 | 0.003775706 | 0.951795672 | 0.011568857 | 0.482906882 | 0.588857398 | 0.582053209 | 0.026312844 | 0.106627835 | 0.01417873 | 0.019492743 | 0.212421445 |
| Cyanobacteria | 0.155654836 | 0.03941974 | 0.196081262 | 0.02155852 | 0.008051807 | 0.008334097 | 0.297372155 | 0.002922025 | 0.03653858 | 0.005955146 | 0.02269849 | 0.096121814 | 0.056825511 | 0.818920129 | 0.011762806 | 0.151265902 | 0.188895024 | 0.015157268 |
| Bacteroidetes | 0.049509908 | 0.166412531 | 0.009558649 | 0.003365676 | 0.138761327 | 0.027456435 | 0.020711674 | 0.001705496 | 0.002333593 | 0.004221593 | 0.020702813 | 0.02537072 | 0.007320376 | 0.005621946 | 0.014917139 | 0.002210106 | 0.001759916 | 0.01711021 |
| Actinobacteria | 0.037320187 | 0.010649975 | 0.011145662 | 0.001749978 | 0.008455013 | 0.005546811 | 0.023208536 | 5.39E-05 | 0.000485035 | 0.002007838 | 0.123132383 | 0.012115626 | 0.002168299 | 0.00024746 | 0.004295042 | 0.001055963 | 0.00328592 | 0.00107637 |
| Tenericutes | 0 | 0 | 9.62E-07 | 0.00034653 | 0 | 0 | 0 | 2.33E-07 | 5.22E-07 | 6.09E-07 | 4.80E-07 | 0.000331438 | 3.58E-06 | 0 | 0 | 0 | 0 | 0 |
| Thaumarchaeota | 0.005700091 | 0.000367854 | 0.000318557 | 0.00021225 | 0.000273934 | 0.005846638 | 0.006794739 | 6.06E-06 | 0.0004035 | 0.000117058 | 0.000904464 | 0.001545904 | 0.000137641 | 4.23E-05 | 0.000225268 | 0.009439953 | 0.019341575 | 0.000353471 |
| Deinococcus-Thermus | 0 | 0 | 9.62E-07 | 0.000433163 | 0.005946518 | 0.000360904 | 2.73E-05 | 0 | 6.52E-07 | 0 | 0.000914065 | 0.003140192 | 3.58E-05 | 0 | 5.80E-05 | 0 | 0.000159124 | 0 |
| Planctomycetes | 0 | 5.93E-05 | 1.73E-05 | 0 | 1.85E-05 | 0 | 4.09E-05 | 0.00032252 | 1.30E-07 | 8.11E-07 | 8.64E-06 | 0.001325751 | 0.000445815 | 0.005823396 | 0.000178146 | 1.04E-06 | 0 | 1.09E-06 |
| Fusobacteria | 0.000173314 | 1.78E-05 | 0.000121264 | 7.36E-05 | 0.000283168 | 5.55E-06 | 0.005457622 | 0 | 8.31E-05 | 0.000188267 | 0.000893902 | 0.000319342 | 0.000100103 | 3.73E-05 | 0.000133897 | 3.13E-06 | 3.18E-06 | 4.63E-05 |
| Euryarchaeota | 0 | 0 | 1.35E-05 | 8.66E-06 | 3.08E-06 | 2.78E-06 | 0 | 1.17E-07 | 0 | 3.85E-06 | 7.83E-05 | 0.000667714 | 0.000786522 | 0.002426104 | 0.000235612 | 3.13E-06 | 3.18E-06 | 1.09E-05 |
| Saccharibacteria | 0 | 0 | 0 | 0 | 0 | 0.000555236 | 0.000682203 | 0 | 0 | 2.01E-05 | 0.002317809 | 0.000246764 | 4.61E-05 | 0 | 5.98E-05 | 0.000104447 | 1.59E-06 | 0 |
| .MHVG. | 0.001752393 | 0.000124596 | 5.00E-05 | 8.66E-06 | 4.31E-05 | 2.78E-06 | 0.001268897 | 2.33E-07 | 0.000112844 | 2.50E-05 | 0.000269803 | 4.23E-05 | 1.11E-05 | 0 | 4.25E-05 | 0.000676819 | 0.001346193 | 4.33E-05 |
| Acidobacteria | 3.85E-05 | 5.93E-06 | 1.06E-05 | 0 | 0.000329336 | 1.39E-05 | 0.001405338 | 0 | 6.52E-06 | 8.52E-06 | 2.59E-05 | 0.000304826 | 0.000187693 | 0.000133056 | 9.37E-05 | 2.09E-06 | 6.36E-06 | 3.02E-05 |
| Verrucomicrobia | 1.93E-05 | 0 | 4.81E-06 | 4.33E-06 | 5.54E-05 | 2.78E-06 | 0 | 0 | 0 | 8.11E-07 | 8.16E-06 | 0.000273376 | 3.72E-05 | 0.000215129 | 8.05E-06 | 0 | 1.59E-06 | 1.09E-06 |
| WSA2 | 0.000924339 | 3.56E-05 | 2.79E-05 | 1.73E-05 | 2.77E-05 | 1.39E-05 | 0.001064236 | 8.16E-07 | 8.24E-05 | 1.16E-05 | 0.000118099 | 1.09E-05 | 5.36E-06 | 2.49E-06 | 1.95E-05 | 0.000284097 | 0.000585578 | 1.60E-05 |
| Crenarchaeota | 0.000924339 | 4.75E-05 | 4.04E-05 | 8.66E-06 | 2.46E-05 | 0 | 0.000886864 | 0 | 6.07E-05 | 1.52E-05 | 0.000136342 | 2.90E-05 | 1.00E-05 | 0 | 4.31E-05 | 0.000465835 | 0.000751067 | 1.66E-05 |
| Thermodesulfobacteria | 0 | 0.000142395 | 9.62E-07 | 0 | 0 | 0 | 0 | 0.000584079 | 0 | 0 | 0 | 4.84E-06 | 7.15E-07 | 0 | 5.75E-07 | 0 | 0 | 0 |
| Synergistetes | 3.85E-05 | 0 | 5.77E-06 | 0 | 3.08E-06 | 2.78E-06 | 0 | 0 | 1.30E-07 | 9.94E-06 | 2.88E-06 | 8.47E-06 | 2.04E-05 | 0 | 2.93E-05 | 2.09E-06 | 0 | 0 |
| Chloroflexi | 3.85E-05 | 7.71E-05 | 5.97E-05 | 0 | 5.85E-05 | 5.55E-06 | 4.09E-05 | 0 | 7.83E-07 | 3.45E-06 | 2.21E-05 | 3.27E-05 | 0.000111186 | 7.46E-06 | 6.21E-05 | 1.04E-06 | 0 | 1.40E-05 |
| Aquificae | 0.000211828 | 1.19E-05 | 7.70E-06 | 0 | 2.46E-05 | 2.78E-06 | 8.19E-05 | 0 | 3.91E-07 | 0.000192527 | 1.39E-05 | 2.78E-05 | 5.72E-06 | 0 | 1.95E-05 | 0 | 0 | 6.12E-06 |
| Elusimicrobia | 0 | 0 | 0 | 0 | 0 | 0.000183228 | 0 | 0 | 0 | 0 | 9.60E-07 | 1.33E-05 | 4.29E-06 | 0 | 1.15E-06 | 0 | 0 | 1.75E-06 |
| Nitrospirae | 1.93E-05 | 1.19E-05 | 8.66E-06 | 1.30E-05 | 3.08E-06 | 2.78E-06 | 0 | 3.26E-06 | 1.30E-07 | 7.30E-06 | 9.12E-06 | 6.41E-05 | 5.93E-05 | 4.97E-05 | 4.42E-05 | 0 | 0 | 3.72E-06 |
| Bathyarchaeota | 0 | 0 | 0 | 0 | 0 | 0 | 0 | 0 | 0 | 0 | 0 | 0 | 0 | 0 | 0 | 0 | 4.77E-06 | 0 |
| Gemmatimonadetes | 0 | 4.75E-05 | 2.60E-05 | 0 | 3.39E-05 | 5.55E-06 | 1.36E-05 | 0 | 5.22E-07 | 1.01E-06 | 3.36E-06 | 1.69E-05 | 2.04E-05 | 1.24E-06 | 0.000113209 | 0 | 1.59E-06 | 0 |
| Fibrobacteres | 0 | 0 | 2.89E-06 | 0 | 0 | 0 | 0 | 0 | 0 | 0 | 1.44E-06 | 0.000107657 | 1.43E-06 | 0 | 1.84E-05 | 0 | 0 | 1.09E-06 |
| Tectomicrobia | 0 | 0 | 0.000102978 | 0 | 0 | 8.33E-06 | 1.36E-05 | 0 | 9.13E-07 | 0 | 4.80E-07 | 1.21E-06 | 0 | 4.97E-06 | 1.72E-06 | 0 | 0 | 0 |
| Nitrospinae | 0 | 0 | 4.81E-06 | 0 | 2.46E-05 | 9.99E-05 | 8.19E-05 | 1.17E-07 | 0 | 0 | 9.60E-07 | 2.42E-06 | 0 | 0 | 0 | 0 | 1.59E-06 | 2.19E-07 |
| Spirochaetes | 0 | 2.97E-05 | 6.74E-06 | 0 | 0 | 0 | 0 | 0 | 0 | 2.03E-07 | 8.64E-06 | 6.77E-05 | 9.80E-05 | 0 | 1.15E-06 | 0 | 1.59E-06 | 0 |
| Chlamydiae | 0 | 0 | 0 | 0 | 0 | 0 | 0 | 0 | 0 | 0 | 0 | 8.35E-05 | 0 | 0 | 0 | 0 | 0 | 0 |
| Atribacteria | 7.70E-05 | 0 | 9.62E-07 | 0 | 0 | 0 | 0 | 0 | 0 | 4.67E-06 | 4.80E-07 | 2.42E-06 | 8.22E-06 | 0 | 5.75E-06 | 0 | 0 | 2.19E-07 |
| Hydrogenedentes | 0 | 0 | 0 | 0 | 2.46E-05 | 5.55E-06 | 0 | 0 | 0 | 6.57E-05 | 1.92E-06 | 7.26E-06 | 1.43E-06 | 1.24E-06 | 5.23E-05 | 0 | 0 | 1.53E-06 |
| Microgenomates | 0 | 0 | 0 | 0 | 0 | 0 | 0 | 0 | 0 | 1.22E-06 | 1.92E-06 | 4.84E-06 | 1.14E-05 | 1.24E-06 | 5.92E-05 | 0 | 0 | 0 |
| Latescibacteria | 0 | 0 | 1.92E-06 | 0 | 0 | 0 | 1.36E-05 | 0 | 1.30E-07 | 0 | 0 | 0 | 3.58E-07 | 0 | 0 | 1.04E-06 | 0 | 0 |
| pMC2A209 | 3.85E-05 | 0 | 0 | 0 | 0 | 0 | 2.73E-05 | 0 | 9.13E-07 | 6.09E-07 | 4.80E-06 | 3.63E-06 | 7.15E-07 | 0 | 2.87E-06 | 1.88E-05 | 4.30E-05 | 4.37E-07 |
| Lentisphaerae | 1.93E-05 | 0 | 1.92E-06 | 0 | 0 | 8.33E-06 | 0 | 0 | 6.65E-06 | 0 | 0 | 2.42E-06 | 3.58E-07 | 0 | 0 | 0 | 0 | 2.19E-07 |
| Ignavibacteriae | 0 | 5.93E-06 | 2.89E-06 | 0 | 3.08E-06 | 0 | 0 | 0 | 6.13E-06 | 0 | 2.40E-06 | 0 | 3.93E-06 | 0 | 5.75E-07 | 0 | 0 | 2.19E-07 |
| Cloacimonetes | 0 | 0 | 0 | 0 | 0 | 0 | 0 | 0 | 0 | 0 | 1.92E-06 | 4.84E-06 | 3.58E-07 | 0 | 1.72E-06 | 0 | 0 | 0 |
| Hadesarchaea | 0 | 0 | 0 | 0 | 0 | 0 | 2.73E-05 | 0 | 2.61E-07 | 2.23E-06 | 1.92E-05 | 0 | 5.36E-06 | 0 | 1.72E-06 | 0 | 7.96E-06 | 1.31E-06 |
| Gracilibacteria | 0 | 0 | 0 | 0 | 0 | 0 | 0 | 0 | 1.30E-07 | 0 | 3.84E-06 | 8.47E-06 | 2.68E-05 | 0 | 2.30E-06 | 0 | 0 | 0 |
| PAUC34f | 0 | 2.37E-05 | 1.54E-05 | 0 | 0 | 0 | 1.36E-05 | 0 | 0 | 0 | 0 | 0 | 0 | 0 | 0 | 0 | 0 | 0 |
| Armatimonadetes | 0 | 0 | 0 | 0 | 0 | 0 | 0 | 0 | 1.30E-07 | 4.06E-07 | 9.60E-07 | 1.21E-06 | 2.18E-05 | 0 | 1.15E-06 | 0 | 0 | 0 |
| Thermotogae | 0 | 0 | 0 | 0 | 0 | 1.94E-05 | 0 | 0 | 1.30E-07 | 0 | 0 | 0 | 7.15E-07 | 0 | 2.30E-06 | 0 | 0 | 0 |
| SR1_(Absconditabacteria) | 1.93E-05 | 0 | 0 | 0 | 0 | 0 | 0 | 0 | 0 | 0 | 0 | 4.84E-06 | 1.07E-06 | 3.73E-06 | 0 | 0 | 0 | 0 |
| AC1 | 0 | 0 | 0 | 0 | 0 | 0 | 0 | 0 | 0 | 0 | 0 | 1.21E-06 | 3.58E-07 | 0 | 1.15E-06 | 0 | 0 | 0 |
| Aminicenantes | 0 | 0 | 0 | 0 | 0 | 0 | 0 | 0 | 0 | 0 | 0 | 3.63E-06 | 8.58E-06 | 0 | 0 | 0 | 0 | 0 |
| Chlorobi | 0 | 0 | 0 | 0 | 0 | 0 | 0 | 0 | 0 | 2.03E-07 | 0 | 9.68E-06 | 2.86E-06 | 0 | 4.60E-06 | 0 | 0 | 5.03E-06 |
| Marinimicrobia_(SAR406_clade) | 0 | 0 | 0 | 0 | 0 | 0 | 0 | 0 | 0 | 0 | 4.80E-07 | 0 | 2.50E-06 | 0 | 0 | 0 | 0 | 0 |
| TM6_(Dependentiae) | 0 | 0 | 0 | 0 | 0 | 0 | 0 | 0 | 1.30E-07 | 0 | 0 | 8.47E-06 | 3.58E-07 | 0 | 4.60E-06 | 0 | 0 | 0 |
| Peregrinibacteria | 0 | 0 | 0 | 0 | 0 | 0 | 0 | 0 | 0 | 0 | 0 | 8.47E-06 | 0 | 0 | 0 | 0 | 0 | 0 |
| Chrysiogenetes | 0 | 0 | 0 | 0 | 0 | 0 | 0 | 0 | 0 | 0 | 0 | 7.26E-06 | 0 | 0 | 0 | 0 | 0 | 0 |
| Parcubacteria | 0 | 0 | 0 | 0 | 0 | 0 | 0 | 0 | 6.39E-06 | 0 | 0 | 0 | 0 | 0 | 1.72E-06 | 0 | 0 | 0 |
| MSBL1 | 0 | 0 | 0 | 0 | 0 | 0 | 0 | 0 | 0 | 1.01E-06 | 5.28E-06 | 0 | 3.22E-06 | 0 | 5.75E-07 | 0 | 0 | 4.37E-07 |
| .DHVEG-6. | 0 | 0 | 0 | 0 | 0 | 0 | 0 | 0 | 0 | 0 | 9.60E-07 | 0 | 7.15E-07 | 0 | 0 | 0 | 0 | 0 |
| WS6 | 0 | 0 | 0 | 0 | 0 | 0 | 0 | 0 | 0 | 0 | 0 | 3.63E-06 | 3.58E-07 | 0 | 1.72E-06 | 0 | 0 | 0 |
| BRC1 | 0 | 0 | 0 | 0 | 0 | 2.78E-06 | 0 | 0 | 7.83E-07 | 0 | 0 | 0 | 0 | 0 | 0 | 0 | 0 | 0 |
| Deferribacteres | 0 | 0 | 1.92E-06 | 0 | 0 | 0 | 0 | 0 | 0 | 0 | 0 | 0 | 0 | 0 | 0 | 0 | 0 | 0 |
| FL0428B-PF49 | 0 | 0 | 0 | 0 | 0 | 0 | 0 | 0 | 0 | 0 | 0 | 2.42E-06 | 0 | 0 | 0 | 0 | 0 | 0 |
| Lokiarchaeota | 0 | 0 | 0 | 0 | 0 | 0 | 0 | 0 | 0 | 0 | 0 | 0 | 0 | 0 | 0 | 2.09E-06 | 1.59E-06 | 0 |
| Omnitrophica | 0 | 0 | 0 | 0 | 0 | 0 | 0 | 0 | 0 | 0 | 0 | 0 | 1.43E-06 | 0 | 0 | 0 | 0 | 0 |
| FBP | 0 | 0 | 0 | 0 | 0 | 0 | 0 | 0 | 0 | 0 | 0 | 0 | 0 | 0 | 0 | 0 | 0 | 1.31E-06 |
| RBG-1_(Zixibacteria) | 0 | 0 | 0 | 0 | 0 | 0 | 0 | 0 | 0 | 0 | 0 | 0 | 0 | 0 | 0 | 0 | 0 | 0 |
| FCPU426 | 0 | 0 | 0 | 0 | 0 | 0 | 0 | 0 | 0 | 0 | 0 | 1.21E-06 | 0 | 0 | 0 | 0 | 0 | 0 |
| Candidatus_Berkelbacteria | 0 | 0 | 0 | 0 | 0 | 0 | 0 | 0 | 0 | 0 | 0 | 0 | 3.58E-07 | 0 | 1.15E-06 | 0 | 0 | 0 |
| SBR1093 | 0 | 0 | 0 | 0 | 0 | 0 | 0 | 0 | 0 | 0 | 0 | 0 | 0 | 0 | 0 | 1.04E-06 | 0 | 0 |
| TVG8AR30 | 0 | 0 | 0 | 0 | 0 | 0 | 0 | 0 | 0 | 0 | 0 | 0 | 0 | 0 | 0 | 1.04E-06 | 0 | 0 |
| Altiarchaeales | 0 | 0 | 0 | 0 | 0 | 0 | 0 | 0 | 0 | 0 | 0 | 0 | 0 | 0 | 0 | 1.04E-06 | 0 | 0 |
| WS1 | 0 | 0 | 0 | 0 | 0 | 0 | 0 | 0 | 0 | 0 | 9.60E-07 | 0 | 0 | 0 | 0 | 0 | 0 | 0 |
| BJ-169 | 0 | 0 | 0 | 0 | 0 | 0 | 0 | 0 | 0 | 0 | 4.80E-07 | 0 | 0 | 0 | 0 | 0 | 0 | 0 |
| Others | 0.000288856 | 2.37E-05 | 1.44E-05 | 1.30E-05 | 2.46E-05 | 5.55E-06 | 0.000382034 | 8.16E-07 | 3.08E-05 | 7.10E-06 | 7.01E-05 | 8.47E-06 | 1.43E-06 | 1.24E-06 | 1.32E-05 | 0.000122203 | 0.000237095 | 8.96E-06 |

^a^RTE, ready-to-eat

**Table. S3.** Relative abundance of bacterial community at the family level in these ready-to-eat food samples

| Taxonomy | Leuconostocaceae | Mitochondria | Moraxellaceae | Enterobacteriaceae | Bacteroidaceae | Vibrionaceae | Lachnospiraceae | Ruminococcaceae | Psychromonadaceae | Pseudomonadaceae | Others |
| --- | --- | --- | --- | --- | --- | --- | --- | --- | --- | --- | --- |
| ^a^RTE meat.1 | 0.003658842 | 0.019160777 | 0.124496909 | 0.051108244 | 0.018968207 | 0.036723218 | 0.007664311 | 0.01003293 | 0 | 0.030695758 | 0.697490805 |
| RTE meat.2 | 0.002307989 | 0.008864102 | 0.072069774 | 0.217579875 | 0 | 0.079652318 | 0.000640778 | 2.97E-05 | 0.108671275 | 0.01050758 | 0.499676644 |
| RTE meat.3 | 0.227525097 | 0.007616507 | 0.009959011 | 0.097743247 | 0.00113853 | 0.008151606 | 0.003824614 | 0.001698652 | 0.000916214 | 0.004967957 | 0.636458565 |
| RTE meat.4 | 0.468504721 | 0.00430564 | 0.01244044 | 0.01560253 | 0 | 0.237100407 | 0.001256173 | 2.60E-05 | 0.001290826 | 0.001949233 | 0.257524041 |
| RTE meat.5 | 0.0133889 | 0.008713558 | 0.281810179 | 0.033983182 | 1.23E-05 | 0.122722348 | 0.002373067 | 0.000273934 | 0 | 0.034518738 | 0.502203782 |
| RTE meat.6 | 9.72E-05 | 0.000274842 | 0.157365071 | 0.088657355 | 0 | 0.614574397 | 0.00030538 | 2.22E-05 | 0 | 0.01413354 | 0.124570039 |
| RTE meat.7 | 0.203378268 | 0.058492059 | 0.045693937 | 0.099356001 | 0.006112536 | 0.010806091 | 0.000545762 | 0.000150085 | 0 | 0.019838454 | 0.555626808 |
| RTE meat.8 | 0.000947511 | 4.18E-05 | 0.005245635 | 0.028590601 | 0 | 0.31792392 | 4.31E-06 | 1.27E-05 | 0.449637227 | 0.001878353 | 0.195717891 |
| RTE vegetables.1 | 0.74016846 | 0.002724308 | 0.002267843 | 0.002172219 | 8.90E-05 | 9.39E-06 | 0.001738976 | 0.00012602 | 0 | 9.35E-05 | 0.250610272 |
| RTE vegetables.2 | 0.006071393 | 0.00119107 | 0.718187521 | 0.118766968 | 4.48E-05 | 0.001825455 | 0.000120913 | 5.94E-05 | 0 | 0.015700982 | 0.138031422 |
| RTE vegetables.3 | 0.422845489 | 0.013127688 | 0.144527466 | 0.12619527 | 0.000506 | 0.006830526 | 0.00175996 | 0.000365818 | 0 | 0.014983663 | 0.268858119 |
| RTE vegetables.4 | 0.21089366 | 0.016039656 | 0.033908269 | 0.115285938 | 0.001351154 | 0.001870084 | 0.003776456 | 0.001478164 | 0.000102818 | 0.056314193 | 0.558979607 |
| RTE vegetables.5 | 0.014009747 | 0.012381646 | 0.052331967 | 0.016114409 | 0.000144434 | 0.019850747 | 0.002655585 | 0.001631676 | 5.76E-05 | 0.207669735 | 0.673152495 |
| RTE vegetables.6 | 0.019371526 | 0.102921024 | 0.006339456 | 0.011180472 | 0 | 5.60E-05 | 0.00022259 | 8.70E-06 | 0 | 0.009720581 | 0.850179688 |
| RTE vegetables.7 | 0.022175151 | 0.011369736 | 0.312942132 | 0.092552579 | 0.000122978 | 0.001663079 | 0.000974056 | 0.000430998 | 3.22E-05 | 0.33019988 | 0.22753723 |
| RTE fruit.1 | 0.002242485 | 0.708981429 | 0.093527397 | 0.012426103 | 0.000312298 | 7.10E-05 | 2.82E-05 | 0.000107581 | 0 | 0.001339015 | 0.180964467 |
| RTE fruit.2 | 0.01548599 | 0.725764951 | 0.002294574 | 0.020380657 | 0 | 3.50E-05 | 0.000128891 | 1.27E-05 | 0 | 0.000631724 | 0.235265475 |
| RTE fruit.3 | 0.161863991 | 0.076072265 | 0.030111679 | 0.630734198 | 5.77E-05 | 0.001792711 | 0.000239363 | 5.25E-05 | 0 | 0.002233838 | 0.096841782 |

^a^RTE, ready-to-eat

**Table. S4.** Relative abundance of bacterial community at the genus level in these ready-to-eat food samples

| Taxonomy | Weissella | Acinetobacter | Bacteroides | Photobacterium | Psychromonas | Leuconostoc | unidentified_Mitochondria | Pseudomonas | Vibrio | Lactobacillus | Others |
| --- | --- | --- | --- | --- | --- | --- | --- | --- | --- | --- | --- |
| ^a^RTE meat.1 | 0.003350729 | 0.120529954 | 0.018968207 | 0.03552928 | 0 | 0.000308113 | 0.01055287 | 0.030406902 | 0.000885825 | 0.02543858 | 0.75402954 |
| RTE meat.2 | 0.001156961 | 0.067151206 | 0 | 0.000409386 | 0.108671275 | 0.001151028 | 0.004610045 | 0.01050758 | 0.076222967 | 0.00444985 | 0.725669702 |
| RTE meat.3 | 0.217426537 | 0.007805139 | 0.00113853 | 0.005123867 | 0.000916214 | 0.010025417 | 0.003736073 | 0.004959295 | 0.00270437 | 0.026677985 | 0.719486574 |
| RTE meat.4 | 0.431218054 | 0.003512952 | 0 | 0.000645413 | 0.001290826 | 0.036740882 | 0.002334748 | 0.001936238 | 0.228857316 | 0.025647579 | 0.267815992 |
| RTE meat.5 | 0.00609118 | 0.260701886 | 1.23E-05 | 0.095818354 | 0 | 0.007214616 | 0.004447577 | 0.034512582 | 0.023192037 | 0.081933911 | 0.486075544 |
| RTE meat.6 | 5.55E-05 | 0.078332736 | 0 | 0.576440769 | 0 | 4.16E-05 | 0.000158242 | 0.01413354 | 0.015974148 | 0.000166571 | 0.314696827 |
| RTE meat.7 | 0.197102003 | 0.034451236 | 0.006112536 | 0.001323473 | 0 | 0.006276265 | 0.030480816 | 0.019770234 | 0.008786771 | 0.02631938 | 0.669377285 |
| RTE meat.8 | 0.000213304 | 0.000175888 | 0 | 0.000677792 | 0.449637227 | 0.000733275 | 2.75E-05 | 0.001831963 | 0.304870676 | 6.67E-05 | 0.241765696 |
| RTE vegetables.1 | 0.737044566 | 0.001610216 | 8.90E-05 | 0 | 0 | 0.003116589 | 0.00114775 | 9.35E-05 | 8.35E-06 | 0.096345777 | 0.160544246 |
| RTE vegetables.2 | 0.000698087 | 0.711143959 | 4.48E-05 | 0.000312019 | 0 | 0.005347743 | 0.000592796 | 0.015697939 | 0.001308534 | 0.000303499 | 0.264550589 |
| RTE vegetables.3 | 0.039408988 | 0.030574143 | 0.000506 | 0.00056505 | 0 | 0.380231992 | 0.006891016 | 0.014930855 | 0.006019197 | 0.012876608 | 0.507996152 |
| RTE vegetables.4 | 0.017673863 | 0.027445231 | 0.001351154 | 2.90E-05 | 0.000102818 | 0.192354914 | 0.011301547 | 0.056296049 | 0.00178299 | 0.249644067 | 0.442018336 |
| RTE vegetables.5 | 0.000873755 | 0.027913671 | 0.000144434 | 0.000122268 | 5.76E-05 | 0.013127055 | 0.008166245 | 0.207608958 | 0.018877604 | 0.29559898 | 0.42750947 |
| RTE vegetables.6 | 0.003162267 | 0.005789821 | 0 | 0 | 0 | 0.016143353 | 0.051393362 | 0.009700685 | 5.60E-05 | 0.000834401 | 0.912920154 |
| RTE vegetables.7 | 0.000985549 | 0.309055102 | 0.000122978 | 4.65E-05 | 3.22E-05 | 0.021059153 | 0.006573012 | 0.330107933 | 0.001595268 | 0.007061476 | 0.323360798 |
| RTE fruit.1 | 0.001898853 | 0.093319546 | 0.000312298 | 0 | 0 | 0.000320653 | 0.340837877 | 0.001295147 | 6.68E-05 | 0.000157716 | 0.561791063 |
| RTE fruit.2 | 0.002227742 | 0.002270706 | 0 | 0 | 0 | 0.009604751 | 0.347082215 | 0.000631724 | 2.86E-05 | 0.001040674 | 0.637113546 |
| RTE fruit.3 | 0.153155759 | 0.02329081 | 5.77E-05 | 3.83E-05 | 0 | 0.008661452 | 0.036342774 | 0.002233401 | 0.001669859 | 0.012841675 | 0.761708306 |

^a^RTE, ready-to-eat

**Table. S5.** Relative abundance of antibiotic resistance genes subtypes in these ready-to-eat food samples

| ARG | ^a^RTE meat.1 | RTE meat.2 | RTE meat.3 | RTE meat.4 | RTE meat.5 | RTE meat.6 | RTE meat.7 | RTE meat.8 | RTE vegetables.1 | RTE vegetables.2 | RTE vegetables.3 | RTE vegetables.4 | RTE vegetables.5 | RTE vegetables.6 | RTE vegetables.7 | RTE fruit.1 | RTE fruit.2 | RTE fruit.3 |
| --- | --- | --- | --- | --- | --- | --- | --- | --- | --- | --- | --- | --- | --- | --- | --- | --- | --- | --- |
| aac2ic | 0 | 0 | 0 | 0 | 0 | 0 | 0 | 6.27E-07 | 0 | 0 | 0 | 0 | 0 | 0 | 0 | 0 | 0 | 0 |
| aac3ia | 0 | 0 | 0 | 0 | 0 | 6.56E-05 | 0 | 0 | 0 | 0 | 0 | 0 | 0 | 0 | 0 | 0 | 0 | 0 |
| aac3iia | 0 | 0 | 0 | 0 | 5.13E-06 | 1.89E-05 | 0 | 0 | 2.90E-06 | 1.34E-06 | 3.22E-06 | 2.05E-06 | 1.82E-06 | 0 | 0 | 0 | 0 | 0 |
| aac3iv | 0 | 0 | 0 | 0 | 0 | 3.15E-05 | 0 | 0 | 0 | 0 | 0 | 0 | 0 | 0 | 0 | 0 | 0 | 8.20E-07 |
| aac6i | 0 | 0 | 0 | 0 | 0 | 0 | 0 | 0 | 0 | 6.55E-07 | 0 | 0 | 0 | 0 | 0 | 7.98E-06 | 0 | 0 |
| aac6ib | 0.000138088 | 0 | 2.31E-06 | 0 | 0 | 5.85E-06 | 3.29E-05 | 0 | 0 | 1.13E-05 | 2.30E-06 | 0 | 1.11E-05 | 0 | 5.93E-06 | 0 | 0 | 0 |
| aac6ic | 0 | 0 | 0 | 0 | 0 | 0 | 0 | 0 | 0 | 0 | 0 | 0 | 0 | 0 | 0 | 0 | 0 | 7.95E-06 |
| aac6ie | 3.86E-05 | 0 | 3.87E-06 | 0 | 0 | 0 | 0 | 0 | 1.19E-05 | 2.21E-06 | 9.62E-07 | 9.82E-06 | 2.17E-06 | 0 | 3.44E-06 | 0 | 0 | 0 |
| aac6ig | 0 | 0 | 0 | 0 | 0 | 0 | 0 | 0 | 0 | 2.64E-06 | 0 | 0 | 0 | 0 | 0 | 0 | 0 | 0 |
| aac6iia | 0 | 0 | 0 | 0 | 0 | 1.47E-05 | 0 | 0 | 0 | 0 | 0 | 0 | 0 | 0 | 2.97E-06 | 0 | 0 | 0 |
| aad9ib | 0 | 0 | 0 | 0 | 0 | 0 | 0 | 0 | 4.91E-07 | 3.69E-07 | 0 | 0 | 1.34E-06 | 0 | 0 | 0 | 0 | 0 |
| aadd | 0 | 0 | 0 | 0 | 0 | 0 | 0 | 0 | 2.96E-06 | 0 | 0 | 2.48E-06 | 0 | 0 | 0 | 0 | 0 | 0 |
| acra | 9.12E-05 | 0.000226942 | 0.000191803 | 3.16E-05 | 4.77E-05 | 0.000150812 | 0.000314797 | 0 | 2.23E-06 | 9.96E-05 | 0.000154645 | 0.000166402 | 2.30E-05 | 8.93E-06 | 7.35E-05 | 0 | 1.56E-05 | 0.000923034 |
| acrb | 9.71E-05 | 0.000537145 | 0.000274455 | 1.01E-05 | 0.000133614 | 0.000269741 | 0.000404223 | 4.35E-06 | 5.51E-06 | 0.000260862 | 0.000332584 | 0.000419553 | 0.000104378 | 1.26E-05 | 0.000231418 | 8.39E-06 | 3.17E-05 | 0.001376096 |
| adea | 0.000256774 | 0 | 9.36E-06 | 0 | 7.58E-06 | 0 | 0 | 0 | 8.08E-07 | 7.36E-05 | 4.80E-06 | 0 | 1.83E-06 | 3.03E-06 | 1.21E-05 | 3.03E-05 | 0 | 2.75E-06 |
| adeb | 0.000214341 | 1.93E-05 | 4.48E-07 | 0 | 5.25E-05 | 1.31E-06 | 1.28E-05 | 0 | 8.65E-07 | 0.000138088 | 9.34E-06 | 1.02E-05 | 1.58E-05 | 1.16E-06 | 3.37E-05 | 3.79E-05 | 0 | 4.81E-06 |
| adec | 7.95E-05 | 0 | 2.99E-06 | 0 | 1.26E-05 | 2.91E-06 | 2.84E-05 | 0 | 0 | 1.45E-05 | 0 | 2.53E-06 | 7.49E-07 | 0 | 9.45E-06 | 2.52E-06 | 0 | 4.56E-07 |
| amrb | 8.88E-06 | 0 | 0 | 0 | 0 | 0 | 0 | 0 | 0 | 9.22E-08 | 0 | 1.13E-06 | 1.00E-06 | 0 | 0 | 0 | 0 | 0 |
| ant2ia | 0.000203437 | 0 | 0 | 0 | 5.89E-06 | 1.21E-05 | 1.97E-05 | 0 | 2.56E-07 | 2.59E-05 | 3.67E-05 | 1.88E-05 | 4.27E-05 | 0 | 7.22E-05 | 0 | 0 | 4.25E-07 |
| ant3ia | 0.000206594 | 9.10E-06 | 1.48E-06 | 0 | 1.88E-05 | 5.19E-05 | 4.21E-05 | 1.82E-07 | 2.04E-06 | 5.46E-05 | 5.10E-06 | 0 | 7.23E-06 | 0 | 1.75E-06 | 0 | 0 | 6.77E-07 |
| ant6ia | 0 | 0 | 1.61E-06 | 0 | 0 | 0 | 0 | 0 | 0 | 0 | 0 | 0 | 0 | 0 | 0 | 0 | 0 | 0 |
| aph33ib | 0.000138317 | 4.26E-05 | 3.47E-06 | 0 | 4.40E-05 | 0.000143473 | 0.000197404 | 8.52E-07 | 1.45E-05 | 3.51E-05 | 0.000109861 | 4.22E-05 | 3.39E-05 | 0 | 1.41E-05 | 6.32E-06 | 0 | 3.83E-06 |
| aph3ia | 0.000256327 | 3.04E-05 | 9.88E-07 | 0 | 5.33E-05 | 6.11E-05 | 0 | 0 | 2.07E-06 | 7.94E-05 | 1.14E-05 | 1.34E-05 | 1.03E-05 | 1.28E-06 | 3.07E-05 | 0.00010112 | 3.80E-06 | 8.47E-06 |
| aph3ib | 0 | 0 | 0 | 0 | 0 | 0 | 0 | 0 | 0 | 0 | 0 | 2.28E-06 | 0 | 0 | 0 | 0 | 0 | 0 |
| aph3iiia | 0 | 0 | 0.001515264 | 0 | 0 | 0 | 0 | 0 | 1.96E-06 | 0 | 0 | 0 | 1.33E-06 | 0 | 2.10E-06 | 0 | 6.82E-06 | 0 |
| aph3via | 0 | 0 | 0 | 0 | 2.27E-05 | 1.04E-05 | 0 | 2.19E-07 | 0 | 0 | 1.78E-06 | 0 | 1.34E-06 | 0 | 1.16E-05 | 0 | 0 | 0 |
| aph6ib | 0 | 0 | 0 | 0 | 0 | 0 | 0 | 0 | 0 | 3.13E-07 | 0 | 0 | 0 | 0 | 0 | 0 | 0 | 0 |
| aph6id | 0.000166685 | 0 | 0 | 1.53E-05 | 3.18E-05 | 7.25E-05 | 0.00014098 | 8.24E-07 | 1.07E-05 | 2.68E-05 | 0.000103075 | 5.08E-05 | 3.11E-05 | 0 | 2.46E-05 | 6.32E-06 | 0 | 7.62E-06 |
| arna | 0 | 0.000121039 | 4.85E-05 | 6.43E-06 | 2.67E-05 | 5.34E-05 | 3.00E-05 | 8.63E-08 | 1.55E-06 | 4.98E-05 | 6.74E-05 | 0.000101606 | 8.27E-05 | 3.63E-06 | 6.70E-05 | 1.78E-06 | 8.11E-06 | 0.000392469 |
| baca | 0.000540453 | 0.000416921 | 0.000942881 | 0.000174729 | 0.000388973 | 0.000196927 | 0.000306764 | 2.05E-05 | 0.000150261 | 0.000355367 | 0.000304336 | 0.000843863 | 0.001043762 | 6.52E-05 | 0.000512035 | 2.75E-05 | 3.52E-05 | 0.001152495 |
| bcr | 2.32E-05 | 0.000143697 | 0.000132624 | 2.19E-05 | 2.95E-05 | 0.000105776 | 0.000169507 | 0 | 1.62E-06 | 6.70E-05 | 0.000112762 | 0.000120606 | 1.89E-05 | 3.09E-06 | 8.35E-05 | 3.02E-06 | 4.48E-06 | 0.000717552 |
| bcra | 0 | 0 | 0 | 0 | 0 | 0 | 0 | 0 | 0 | 0 | 2.17E-06 | 0 | 0 | 0 | 0 | 0 | 0 | 3.33E-07 |
| bl1_acc | 0 | 0 | 0 | 0 | 0 | 3.47E-06 | 0 | 0 | 0 | 0 | 0 | 0 | 0 | 0 | 0 | 0 | 0 | 0 |
| bl1_ampc | 0 | 5.28E-05 | 0.000158128 | 1.11E-05 | 7.71E-06 | 4.67E-05 | 0.000174267 | 0 | 2.18E-06 | 4.86E-05 | 7.93E-05 | 4.33E-05 | 0 | 0 | 1.59E-05 | 0 | 4.68E-06 | 0.000171465 |
| bl1_asba | 0 | 0 | 0 | 0 | 0 | 0 | 0 | 0 | 0 | 1.01E-06 | 0 | 0 | 4.56E-07 | 0 | 0 | 0 | 0 | 2.77E-07 |
| bl1_ceps | 0 | 0 | 0 | 0 | 3.85E-06 | 0 | 0 | 0 | 0 | 5.95E-06 | 0 | 0 | 0 | 0 | 3.38E-06 | 0 | 0 | 0 |
| bl1_cmy2 | 0 | 0 | 1.22E-06 | 0 | 0 | 9.56E-05 | 0 | 0 | 3.36E-07 | 0 | 4.75E-06 | 1.38E-05 | 2.29E-06 | 0 | 5.78E-06 | 0 | 0 | 1.08E-06 |
| bl1_ec | 0 | 0 | 2.46E-06 | 0 | 0 | 0 | 8.39E-05 | 0 | 0 | 1.17E-06 | 1.22E-06 | 7.63E-06 | 0 | 0 | 2.91E-06 | 0 | 0 | 2.81E-07 |
| bl1_fox | 0.000192836 | 0 | 0 | 0 | 3.58E-06 | 0 | 0 | 0 | 0 | 7.39E-06 | 0 | 0 | 6.23E-06 | 0 | 1.44E-06 | 0 | 0 | 0 |
| bl1_mox | 4.84E-05 | 7.46E-06 | 2.43E-06 | 0 | 7.69E-06 | 0 | 0 | 0 | 0 | 6.16E-05 | 6.03E-07 | 0 | 2.73E-06 | 0 | 3.59E-06 | 0 | 0 | 8.32E-07 |
| bl1_pao | 0 | 0 | 0 | 0 | 0 | 0 | 0 | 0 | 0 | 0 | 0 | 0 | 4.39E-07 | 0 | 0 | 0 | 0 | 0 |
| bl1_pse | 0 | 0 | 0 | 0 | 0 | 0 | 0 | 0 | 0 | 0 | 0 | 0 | 8.93E-07 | 0 | 2.81E-06 | 0 | 0 | 0 |
| bl1_sm | 0 | 0 | 0 | 0 | 0 | 0 | 0 | 0 | 0 | 5.08E-07 | 1.23E-06 | 1.55E-06 | 0 | 0 | 0 | 0 | 0 | 3.10E-06 |
| bl2_len | 0 | 0 | 0 | 0 | 0 | 0 | 0 | 0 | 0 | 0 | 1.63E-06 | 0 | 0 | 0 | 0 | 0 | 0 | 7.50E-07 |
| bl2_veb | 0 | 0 | 0 | 0 | 0 | 0 | 0 | 0 | 0 | 0 | 0 | 0 | 5.82E-07 | 0 | 0 | 0 | 0 | 0 |
| bl2a_1 | 0 | 0 | 0 | 0 | 0 | 0 | 0 | 0 | 0 | 0 | 3.59E-06 | 0 | 0 | 0 | 0 | 0 | 0 | 4.01E-07 |
| bl2a_iii2 | 0 | 0 | 3.02E-06 | 6.90E-06 | 0 | 0 | 0 | 0 | 0 | 0 | 0 | 0 | 0 | 0 | 0 | 0 | 0 | 0 |
| bl2a_nps | 3.15E-05 | 0 | 0 | 0 | 1.00E-05 | 0 | 0 | 0 | 0 | 0 | 0 | 0 | 0 | 0 | 0 | 0 | 0 | 0 |
| bl2a_okp | 0 | 0 | 1.62E-06 | 0 | 0 | 0 | 0 | 0 | 4.47E-07 | 0 | 0 | 0 | 0 | 4.19E-06 | 3.83E-06 | 0 | 0 | 1.48E-06 |
| bl2a_pc | 0 | 0 | 0 | 0 | 0 | 0 | 0 | 0 | 3.27E-06 | 0 | 8.95E-07 | 0 | 0 | 0 | 0 | 0 | 0 | 0 |
| bl2b_tem | 0.000742669 | 0 | 4.86E-06 | 1.48E-05 | 1.03E-05 | 0 | 0 | 0 | 3.57E-06 | 6.71E-07 | 0 | 1.44E-05 | 0 | 0 | 0 | 0 | 0 | 7.03E-06 |
| bl2b_tem1 | 0 | 0 | 0 | 0 | 0 | 0 | 0 | 0 | 6.70E-07 | 0 | 8.04E-07 | 0 | 0 | 0 | 0 | 0 | 0 | 4.07E-06 |
| bl2be_ctxm | 0.000126948 | 0 | 1.29E-05 | 0 | 0 | 0 | 0 | 0 | 0 | 3.76E-05 | 8.87E-06 | 3.45E-05 | 1.20E-06 | 0 | 2.49E-05 | 0 | 0 | 7.28E-07 |
| bl2be_oxy1 | 0 | 9.82E-06 | 0 | 0 | 0 | 0 | 0 | 0 | 0 | 1.36E-06 | 1.19E-05 | 4.04E-06 | 6.00E-07 | 0 | 0 | 0 | 0 | 9.10E-06 |
| bl2be_shv2 | 6.97E-05 | 0 | 1.91E-05 | 0 | 0 | 0 | 0 | 0 | 0 | 3.35E-07 | 2.05E-05 | 2.17E-05 | 0 | 0 | 2.07E-06 | 0 | 0 | 0.000124169 |
| bl2c_bro | 0 | 0 | 0 | 0 | 9.85E-06 | 0 | 0 | 3.82E-07 | 2.36E-06 | 0 | 1.54E-06 | 0 | 8.17E-06 | 2.01E-06 | 0 | 0 | 0 | 0 |
| bl2c_pse1 | 0 | 0 | 0 | 0 | 0 | 0 | 0 | 0 | 0 | 1.26E-06 | 0 | 0 | 0 | 1.97E-06 | 0 | 0 | 0 | 0 |
| bl2d_oxa1 | 0 | 0 | 0 | 0 | 0 | 0 | 0 | 0 | 0 | 1.39E-06 | 0 | 0 | 6.30E-07 | 0 | 0 | 0 | 0 | 0 |
| bl2d_oxa10 | 6.94E-05 | 0 | 0 | 0 | 0 | 0 | 0 | 0 | 0 | 5.77E-06 | 0 | 0 | 1.31E-06 | 0 | 0 | 0 | 0 | 0 |
| bl2d_oxa2 | 0 | 0 | 0 | 0 | 0 | 0 | 0 | 0 | 0 | 1.40E-06 | 0 | 0 | 0 | 0 | 1.99E-06 | 0 | 0 | 0 |
| bl2e_cbla | 0 | 0 | 0 | 0 | 0 | 0 | 0 | 0 | 2.16E-07 | 0 | 0 | 0 | 0 | 0 | 0 | 0 | 0 | 0 |
| bl2e_cfxa | 0 | 0 | 4.33E-06 | 0 | 0 | 0 | 0 | 0 | 1.99E-07 | 0 | 0 | 0 | 1.08E-06 | 0 | 0 | 3.64E-06 | 0 | 0 |
| bl2e_fpm | 0 | 0 | 0 | 0 | 0 | 0 | 0 | 0 | 0 | 0 | 0 | 4.33E-06 | 0 | 0 | 0 | 0 | 0 | 0 |
| bl3_cpha | 0.00054513 | 0 | 0 | 0 | 0 | 0 | 0 | 0 | 2.51E-07 | 2.34E-05 | 1.81E-06 | 0 | 6.16E-06 | 0 | 3.24E-06 | 0 | 0 | 0 |
| bl3_l | 0 | 0 | 0 | 0 | 0 | 0 | 0 | 0 | 0 | 0 | 1.59E-06 | 2.02E-06 | 0 | 0 | 0 | 0 | 0 | 7.30E-07 |
| ble | 0 | 0 | 0 | 0 | 0 | 0 | 0 | 0 | 1.92E-06 | 0 | 0 | 0 | 2.61E-06 | 0 | 0 | 0 | 0 | 0 |
| blt | 0 | 0 | 0 | 2.12E-05 | 0 | 0 | 0 | 0 | 0 | 0 | 0 | 2.94E-06 | 0 | 0 | 0 | 0 | 0 | 0 |
| bmr | 0 | 0 | 2.38E-06 | 0 | 0 | 0 | 0 | 0 | 0 | 0 | 0 | 0 | 1.79E-06 | 0 | 0 | 0 | 0 | 8.17E-07 |
| cata1 | 0.02739931 | 0 | 6.13E-06 | 0 | 0 | 0 | 0 | 0 | 0 | 1.71E-06 | 0 | 0 | 0 | 0 | 0 | 0 | 0 | 0 |
| cata2 | 0 | 0 | 0 | 0 | 0 | 0 | 0 | 0 | 0 | 3.15E-06 | 0 | 0 | 0 | 0 | 0 | 0 | 0 | 0 |
| cata4 | 0 | 0 | 0 | 0 | 0 | 0 | 0 | 0 | 0 | 0 | 0 | 0 | 1.60E-06 | 0 | 0 | 0 | 0 | 0 |
| cata7 | 0 | 0 | 0 | 0 | 1.36E-05 | 0 | 0 | 0 | 0 | 0 | 3.21E-06 | 0 | 0 | 0 | 0 | 0 | 4.14E-06 | 0 |
| cata8 | 0 | 0 | 0 | 0 | 0 | 0 | 0 | 0 | 2.38E-06 | 0 | 0 | 1.64E-05 | 4.92E-06 | 0 | 0 | 0 | 0 | 0 |
| catb1 | 0 | 0 | 0 | 0 | 1.40E-05 | 0 | 0 | 0 | 0 | 0 | 0 | 1.12E-05 | 0 | 0 | 0 | 0 | 0 | 1.01E-06 |
| catb2 | 0 | 0 | 0 | 0 | 0 | 0 | 0 | 0 | 0 | 0 | 1.09E-06 | 0 | 0 | 0 | 0 | 0 | 0 | 1.51E-06 |
| catb3 | 0 | 0 | 0 | 0 | 0 | 0 | 0 | 0 | 3.04E-07 | 2.28E-06 | 1.09E-06 | 2.79E-06 | 0 | 0 | 0 | 0 | 0 | 1.01E-06 |
| catb5 | 0 | 0 | 0 | 0 | 0 | 0 | 0 | 0 | 0 | 4.58E-07 | 0 | 0 | 0 | 0 | 0 | 0 | 0 | 0 |
| ceob | 2.32E-05 | 0 | 0 | 7.48E-06 | 2.06E-05 | 0 | 0 | 0 | 5.68E-07 | 5.34E-06 | 7.98E-07 | 4.17E-06 | 1.21E-06 | 1.14E-06 | 1.34E-05 | 1.08E-05 | 0 | 1.08E-06 |
| cml_e1 | 6.67E-05 | 0 | 0 | 0 | 0 | 0 | 3.16E-05 | 0 | 1.53E-07 | 5.95E-06 | 0 | 0 | 0 | 0 | 2.62E-06 | 0 | 0 | 0 |
| cml_e3 | 2.29E-05 | 4.23E-05 | 0 | 0.000110213 | 0 | 1.01E-05 | 6.53E-05 | 8.81E-05 | 9.50E-07 | 6.66E-06 | 3.99E-06 | 8.73E-06 | 7.33E-06 | 0 | 2.72E-06 | 4.35E-06 | 0 | 5.25E-07 |
| cml_e4 | 0 | 0 | 9.76E-07 | 0 | 0 | 0 | 0 | 0 | 1.75E-06 | 0 | 0 | 0 | 3.67E-07 | 0 | 0 | 0 | 0 | 0 |
| cml_e8 | 0 | 0 | 0 | 0 | 0 | 6.93E-06 | 1.69E-05 | 0 | 1.64E-06 | 0 | 2.36E-06 | 1.50E-06 | 0 | 0 | 0 | 0 | 0 | 1.08E-06 |
| dfra1 | 0 | 0 | 0 | 0 | 1.93E-05 | 0 | 0 | 3.73E-07 | 0 | 0 | 1.46E-06 | 3.85E-06 | 4.56E-06 | 0 | 0 | 0 | 0 | 0 |
| dfra12 | 0 | 0 | 0 | 0 | 0 | 0 | 0 | 0 | 0 | 1.74E-06 | 0 | 0 | 0 | 0 | 0 | 0 | 0 | 0 |
| dfra14 | 0 | 0 | 0 | 0 | 0 | 0 | 0 | 3.54E-07 | 0 | 9.74E-06 | 0 | 0 | 1.10E-06 | 0 | 0 | 0 | 0 | 0 |
| dfra16 | 0 | 0 | 0 | 0 | 0 | 0 | 0.00017289 | 0 | 0 | 0 | 0 | 0 | 0 | 0 | 0 | 0 | 0 | 0 |
| dfra17 | 0 | 0 | 0 | 0 | 0 | 0 | 0 | 7.36E-07 | 0 | 0 | 0 | 0 | 0 | 0 | 0 | 0 | 0 | 0 |
| dfrb2 | 0 | 0 | 0 | 0 | 0 | 0 | 0 | 0 | 0 | 0 | 0 | 1.49E-05 | 0 | 0 | 0 | 0 | 0 | 0 |
| emea | 0 | 2.21E-05 | 0.000870181 | 0 | 1.52E-05 | 2.10E-05 | 3.44E-05 | 5.93E-07 | 4.97E-07 | 1.98E-06 | 4.10E-05 | 0.000226265 | 9.90E-06 | 0 | 0.000150333 | 0 | 4.61E-06 | 2.46E-06 |
| emrd | 7.04E-05 | 0.000499082 | 0.00023432 | 5.38E-06 | 2.61E-05 | 0.000231081 | 0.00018606 | 1.44E-07 | 1.31E-06 | 0.000129722 | 0.000195257 | 0.000138535 | 2.22E-05 | 0 | 9.07E-05 | 5.94E-06 | 2.33E-05 | 0.001479595 |
| erea | 0 | 0 | 0 | 0 | 0 | 0 | 0 | 0 | 0 | 9.46E-07 | 0 | 0 | 0 | 0 | 0 | 0 | 0 | 0 |
| ermb | 0 | 0 | 0.001750935 | 0 | 0 | 0 | 0 | 0 | 2.30E-06 | 0 | 3.75E-06 | 7.04E-06 | 2.29E-05 | 0 | 3.29E-06 | 0 | 0 | 0 |
| ermc | 0 | 2.33E-05 | 3.79E-06 | 0 | 0 | 0 | 0 | 0 | 0 | 0 | 0 | 0 | 5.70E-06 | 0 | 0 | 0 | 0 | 0 |
| ermf | 6.94E-05 | 0 | 1.04E-05 | 0 | 0 | 0 | 0 | 0 | 4.80E-07 | 0 | 1.73E-06 | 0 | 0 | 0 | 0 | 0 | 0 | 0 |
| ermt | 0 | 0 | 0 | 0 | 0 | 0 | 0 | 0 | 0 | 3.93E-07 | 0 | 0 | 7.18E-07 | 0 | 0 | 0 | 0 | 0 |
| fosa | 0 | 4.02E-05 | 4.58E-05 | 0 | 0 | 1.91E-05 | 0.000186283 | 0 | 9.03E-07 | 1.90E-05 | 6.99E-05 | 1.66E-05 | 2.46E-06 | 0 | 1.36E-05 | 2.07E-05 | 1.26E-05 | 5.69E-05 |
| fosb | 0 | 0 | 3.00E-06 | 0 | 0 | 0 | 0 | 0 | 4.58E-07 | 0 | 4.88E-06 | 0 | 0 | 0 | 0 | 0 | 0 | 0 |
| ksga | 0 | 0.000521534 | 0.000210208 | 0 | 6.99E-05 | 0.000123937 | 0.000120676 | 1.55E-05 | 2.11E-06 | 0.000141303 | 0.00019302 | 0.000174389 | 4.79E-05 | 4.40E-06 | 0.000139628 | 8.57E-06 | 1.63E-05 | 0.001020543 |
| lmrb | 0 | 0 | 9.72E-07 | 0 | 0 | 0 | 0 | 0 | 0 | 0 | 6.76E-06 | 0 | 1.46E-06 | 0 | 0 | 0 | 0 | 0 |
| lmrp | 0 | 1.40E-05 | 9.09E-06 | 0 | 0 | 0 | 0 | 0 | 3.14E-07 | 1.41E-06 | 2.31E-05 | 0.000262159 | 1.54E-05 | 0 | 0.000113015 | 0 | 0 | 2.86E-06 |
| lnua | 0.00011441 | 0 | 1.43E-05 | 5.25E-05 | 0 | 0 | 0.000571498 | 0 | 0.000170601 | 2.38E-06 | 0.00015817 | 6.91E-05 | 2.80E-05 | 4.82E-05 | 1.36E-05 | 0 | 0 | 8.26E-05 |
| lnub | 0 | 0 | 1.39E-05 | 0 | 0 | 0 | 0 | 0 | 4.79E-07 | 0 | 0 | 0 | 0 | 0 | 0 | 0 | 0 | 0 |
| lsa | 0 | 0 | 0.001103984 | 0 | 0 | 0 | 0 | 0 | 0 | 0 | 4.63E-07 | 0 | 0 | 0 | 0 | 0 | 0 | 0 |
| macb | 0 | 0.000220688 | 0.000152149 | 0 | 7.73E-05 | 0.000151093 | 0.000234928 | 1.54E-05 | 1.29E-06 | 8.70E-05 | 9.77E-05 | 0.000108265 | 3.85E-05 | 4.63E-06 | 7.31E-05 | 5.43E-06 | 1.93E-05 | 0.000598746 |
| mdfa | 0 | 0.000132093 | 0.000103184 | 0 | 8.62E-05 | 0.000168607 | 9.64E-05 | 0 | 2.81E-06 | 5.83E-05 | 9.99E-05 | 0.000110387 | 1.32E-05 | 2.92E-06 | 5.29E-05 | 1.42E-06 | 4.34E-06 | 0.000604961 |
| mdr | 0 | 0 | 0 | 0 | 0 | 0 | 2.55E-05 | 0 | 0 | 3.80E-07 | 8.14E-06 | 0 | 5.11E-06 | 0 | 0 | 0 | 0 | 8.19E-07 |
| mdte | 0 | 0 | 1.20E-06 | 0 | 0 | 0 | 3.50E-05 | 0 | 0 | 0 | 3.74E-06 | 4.71E-06 | 0 | 0 | 0 | 0 | 0 | 0 |
| mdtf | 2.68E-05 | 5.51E-06 | 1.34E-06 | 0 | 0 | 5.23E-06 | 5.09E-05 | 0 | 0 | 2.13E-06 | 5.11E-06 | 8.50E-06 | 6.72E-07 | 5.79E-07 | 9.01E-06 | 0 | 1.72E-06 | 2.15E-06 |
| mdtg | 0 | 0.000153773 | 8.62E-05 | 1.05E-05 | 4.68E-05 | 0.00012932 | 0.000225519 | 2.77E-07 | 1.56E-06 | 5.25E-05 | 0.000101637 | 0.000113669 | 2.85E-05 | 4.42E-06 | 7.21E-05 | 4.26E-06 | 0 | 0.000504293 |
| mdth | 0 | 0.000177814 | 9.37E-05 | 0 | 3.21E-05 | 0.000114396 | 0.000326588 | 2.83E-07 | 2.28E-06 | 4.18E-05 | 7.97E-05 | 7.54E-05 | 1.48E-05 | 2.98E-06 | 3.49E-05 | 5.82E-06 | 0 | 0.000514056 |
| mdtk | 2.02E-05 | 0.000187549 | 9.47E-05 | 0 | 6.77E-05 | 8.63E-05 | 0.000145026 | 0 | 2.25E-06 | 7.23E-05 | 8.09E-05 | 0.000115871 | 1.15E-05 | 1.31E-06 | 3.92E-05 | 3.84E-06 | 0 | 0.000407851 |
| mdtl | 0 | 4.38E-05 | 2.02E-05 | 0 | 3.77E-06 | 3.47E-05 | 5.07E-05 | 0 | 6.56E-07 | 3.96E-05 | 3.78E-05 | 2.86E-05 | 3.57E-06 | 0 | 3.17E-05 | 0 | 4.57E-06 | 0.000386418 |
| mdtm | 0 | 0 | 6.71E-06 | 0 | 0 | 0 | 3.22E-05 | 0 | 0 | 1.64E-06 | 1.45E-05 | 1.72E-05 | 2.53E-06 | 0 | 3.31E-06 | 0 | 2.16E-06 | 0.000155927 |
| mdtn | 0 | 1.71E-05 | 0 | 0 | 0 | 7.90E-06 | 3.96E-05 | 0 | 5.59E-07 | 3.83E-05 | 8.26E-06 | 1.37E-05 | 0 | 0 | 2.74E-05 | 0 | 0 | 0 |
| mdto | 0 | 0 | 3.40E-06 | 0 | 0 | 0 | 5.81E-05 | 0 | 0 | 2.68E-06 | 3.72E-06 | 2.59E-06 | 0 | 0 | 6.04E-06 | 0 | 0 | 1.55E-07 |
| mdtp | 0 | 0 | 6.67E-06 | 0 | 0 | 0 | 1.35E-05 | 0 | 2.62E-07 | 6.76E-06 | 4.26E-06 | 1.93E-05 | 0 | 0 | 7.90E-06 | 0 | 0 | 0 |
| meca | 0 | 0 | 1.74E-05 | 0 | 1.33E-05 | 0 | 1.99E-05 | 0 | 5.75E-07 | 2.89E-07 | 5.19E-06 | 8.84E-06 | 4.98E-06 | 0 | 0 | 3.52E-06 | 2.68E-06 | 9.56E-07 |
| mecr1 | 0 | 0 | 0 | 0 | 0 | 0 | 0 | 0 | 0 | 0 | 0 | 0 | 0 | 0 | 0 | 0 | 0 | 3.63E-07 |
| mefa | 0 | 0 | 4.58E-06 | 0 | 0 | 0 | 3.26E-05 | 0 | 2.21E-06 | 9.48E-07 | 0 | 0 | 0 | 0 | 0 | 0 | 0 | 0 |
| mepa | 0 | 0 | 3.08E-06 | 0 | 1.30E-05 | 0 | 2.93E-05 | 0 | 1.82E-05 | 0 | 1.02E-06 | 0 | 0 | 0 | 0 | 0 | 1.19E-05 | 0 |
| mexa | 0 | 0 | 2.26E-06 | 0 | 0 | 0 | 1.49E-05 | 0 | 0 | 1.00E-06 | 0 | 3.07E-06 | 9.09E-06 | 0 | 7.17E-07 | 3.06E-06 | 0 | 5.53E-07 |
| mexb | 0.000141349 | 2.74E-05 | 1.29E-05 | 6.09E-06 | 5.20E-05 | 1.68E-05 | 1.89E-05 | 1.25E-06 | 3.67E-07 | 2.37E-05 | 2.69E-05 | 0.000226376 | 0.000545656 | 9.73E-06 | 0.000324775 | 5.59E-07 | 0 | 3.36E-05 |
| mexc | 0 | 0 | 0 | 0 | 0 | 0 | 0 | 0 | 0 | 0 | 0 | 0 | 9.05E-07 | 0 | 0 | 0 | 0 | 0 |
| mexd | 0 | 0 | 4.44E-06 | 0 | 2.82E-06 | 0 | 6.31E-06 | 0 | 0 | 2.03E-06 | 5.52E-06 | 2.09E-05 | 5.62E-05 | 0 | 1.27E-05 | 5.62E-07 | 0 | 2.04E-07 |
| mexe | 0 | 0 | 2.24E-06 | 0 | 0 | 0 | 0 | 0 | 0 | 2.32E-07 | 5.56E-07 | 1.42E-06 | 2.10E-06 | 0 | 2.65E-06 | 0 | 0 | 0 |
| mexf | 0.000166103 | 5.38E-06 | 6.56E-06 | 6.01E-06 | 8.19E-05 | 2.18E-05 | 1.87E-05 | 4.35E-06 | 1.21E-07 | 2.52E-05 | 2.87E-05 | 0.000304473 | 0.000735156 | 2.49E-05 | 0.000446054 | 1.11E-06 | 0 | 1.00E-06 |
| mexi | 9.00E-06 | 0 | 4.51E-07 | 0 | 2.86E-06 | 5.28E-06 | 0 | 0 | 0 | 5.61E-07 | 1.34E-06 | 5.15E-06 | 6.10E-06 | 0 | 1.07E-05 | 1.14E-06 | 0 | 8.25E-07 |
| mexw | 0.000109474 | 1.12E-05 | 1.83E-06 | 0 | 3.49E-05 | 4.03E-06 | 5.20E-05 | 3.96E-07 | 1.26E-07 | 1.99E-05 | 2.37E-05 | 0.000200501 | 0.000368068 | 5.34E-06 | 0.000370968 | 2.31E-06 | 0 | 1.15E-06 |
| mexx | 0 | 0 | 0 | 0 | 0 | 0 | 0 | 0 | 0 | 0 | 1.16E-06 | 0 | 0 | 0 | 0 | 0 | 0 | 0 |
| mexy | 0 | 0 | 0 | 0 | 0 | 0 | 1.26E-05 | 0 | 0 | 9.20E-08 | 0 | 5.63E-07 | 1.00E-06 | 0 | 0 | 0 | 0 | 0 |
| mfpa | 0 | 3.11E-05 | 0 | 0 | 0 | 0 | 0 | 0 | 0 | 0 | 0 | 0 | 0 | 0 | 0 | 0 | 0 | 0 |
| mphb | 0 | 0 | 0 | 0 | 0 | 0 | 0 | 0 | 2.12E-07 | 0 | 0 | 0 | 0 | 0 | 0 | 0 | 0 | 0 |
| mphc | 0 | 0 | 0 | 0 | 0 | 0 | 0 | 0 | 1.92E-06 | 3.21E-07 | 0 | 0 | 5.82E-07 | 0 | 0 | 0 | 0 | 0 |
| msra | 0 | 0 | 0 | 0 | 0 | 0 | 0 | 0 | 5.25E-07 | 0 | 9.44E-07 | 0 | 1.43E-06 | 0 | 0 | 0 | 0 | 0 |
| nora | 0 | 0 | 1.20E-05 | 1.10E-05 | 3.81E-06 | 0 | 6.84E-05 | 0 | 1.80E-05 | 7.46E-07 | 2.38E-06 | 0 | 3.16E-06 | 0 | 0 | 0 | 4.61E-06 | 1.64E-06 |
| norm | 0 | 1.88E-05 | 0 | 6.05E-05 | 6.45E-06 | 5.94E-06 | 0 | 6.24E-05 | 0 | 0 | 2.02E-06 | 2.58E-06 | 7.64E-06 | 0 | 0 | 0 | 0 | 0 |
| opcm | 0 | 0 | 0 | 0 | 0 | 0 | 2.58E-05 | 0 | 0 | 0 | 0 | 0 | 0 | 0 | 0 | 0 | 0 | 0 |
| opmd | 0 | 0 | 0 | 0 | 0 | 0 | 2.71E-05 | 0 | 0 | 0 | 9.46E-07 | 0 | 3.58E-07 | 0 | 0 | 0 | 0 | 0 |
| oprj | 0 | 0 | 0 | 0 | 0 | 0 | 0 | 0 | 0 | 0 | 0 | 1.23E-06 | 6.55E-06 | 0 | 1.72E-06 | 0 | 0 | 2.21E-07 |
| oprm | 0 | 0 | 0 | 0 | 0 | 2.79E-06 | 0 | 0 | 0 | 0 | 0 | 0 | 1.15E-05 | 0 | 3.96E-06 | 0 | 0 | 0 |
| oprn | 0 | 0 | 1.96E-06 | 0 | 0 | 0 | 0 | 0 | 0 | 1.22E-06 | 9.76E-07 | 8.72E-06 | 5.91E-06 | 1.27E-06 | 5.82E-06 | 0 | 0 | 0 |
| pbp2 | 0 | 1.79E-05 | 8.70E-06 | 0 | 0 | 1.05E-05 | 3.12E-05 | 1.77E-07 | 9.93E-08 | 4.47E-05 | 1.85E-05 | 5.50E-05 | 5.02E-06 | 9.78E-07 | 2.83E-05 | 9.08E-07 | 1.38E-06 | 4.67E-05 |
| pbp2b | 0 | 0 | 7.98E-07 | 0 | 0 | 0 | 1.11E-05 | 0 | 0 | 0 | 0 | 0 | 5.99E-07 | 0 | 0 | 0 | 9.21E-06 | 0 |
| qac | 0 | 0 | 4.30E-06 | 0 | 0 | 1.26E-05 | 0 | 0 | 0 | 0 | 0 | 0 | 0 | 0 | 0 | 0 | 0 | 0 |
| qaca | 0 | 0 | 0 | 0 | 0 | 0 | 0 | 0 | 0 | 0 | 0 | 0 | 6.78E-07 | 0 | 0 | 0 | 3.47E-06 | 0 |
| qacb | 0 | 0 | 1.80E-06 | 0 | 0 | 0 | 0 | 0 | 4.98E-07 | 0 | 0 | 0 | 6.78E-07 | 0 | 0 | 0 | 3.47E-06 | 0 |
| qnra | 0 | 1.30E-05 | 2.12E-05 | 9.71E-06 | 6.73E-06 | 0 | 0 | 0.000141224 | 0 | 0 | 0 | 0 | 1.04E-05 | 0 | 0 | 0 | 0 | 0 |
| qnrb | 0 | 0 | 0 | 0 | 0 | 5.05E-05 | 6.15E-05 | 0 | 0 | 6.69E-06 | 6.39E-06 | 2.45E-05 | 3.25E-06 | 0 | 5.11E-06 | 0 | 0 | 3.93E-06 |
| qnrs | 0.000253896 | 0 | 2.12E-06 | 0 | 0 | 0 | 0 | 0 | 0 | 1.36E-05 | 3.16E-06 | 0 | 4.38E-05 | 0 | 1.13E-05 | 0 | 0 | 9.70E-07 |
| rosa | 2.28E-05 | 9.85E-05 | 4.12E-05 | 5.25E-06 | 4.76E-05 | 2.67E-05 | 6.62E-05 | 0 | 1.10E-06 | 2.07E-05 | 3.93E-05 | 7.19E-05 | 1.77E-05 | 6.02E-06 | 3.68E-05 | 0 | 2.20E-06 | 0.000162536 |
| rosb | 1.70E-05 | 0.000104146 | 3.53E-05 | 7.81E-06 | 1.33E-05 | 6.34E-05 | 3.60E-05 | 0 | 4.59E-07 | 6.16E-05 | 5.75E-05 | 0.000146102 | 3.05E-05 | 1.08E-05 | 4.81E-05 | 3.12E-06 | 3.17E-06 | 0.000282462 |
| smea | 4.65E-05 | 0 | 0 | 0 | 0 | 0 | 0 | 0 | 0 | 0 | 2.31E-06 | 0 | 0 | 0 | 0 | 0 | 0 | 0 |
| smeb | 7.06E-05 | 0 | 6.19E-06 | 2.02E-06 | 1.68E-05 | 3.88E-06 | 3.78E-05 | 0 | 0 | 8.07E-06 | 5.28E-06 | 2.24E-06 | 3.49E-06 | 2.86E-06 | 9.17E-06 | 1.68E-06 | 0 | 6.17E-06 |
| smec | 3.93E-05 | 0 | 4.92E-06 | 1.35E-05 | 6.24E-06 | 0 | 0 | 0 | 0 | 0 | 1.96E-06 | 0 | 7.40E-07 | 0 | 0 | 0 | 0 | 6.75E-07 |
| smed | 0.000164408 | 4.35E-05 | 3.53E-06 | 0 | 3.73E-06 | 0 | 6.71E-05 | 0 | 0 | 0 | 2.34E-06 | 4.48E-06 | 0 | 0 | 0 | 1.49E-06 | 0 | 0 |
| smee | 1.78E-05 | 0 | 1.38E-05 | 0 | 2.55E-05 | 1.04E-05 | 2.54E-05 | 1.10E-07 | 0 | 5.92E-06 | 4.88E-06 | 2.83E-06 | 1.09E-05 | 5.77E-07 | 1.32E-06 | 5.64E-07 | 1.72E-06 | 2.96E-06 |
| smef | 0 | 0 | 8.04E-06 | 0 | 1.26E-05 | 0 | 5.69E-05 | 0 | 0 | 0 | 2.46E-06 | 0 | 0 | 0 | 0 | 0 | 1.91E-06 | 4.50E-07 |
| str | 0 | 0 | 0.000735895 | 1.65E-05 | 0 | 0 | 4.67E-05 | 0 | 3.87E-06 | 0 | 1.63E-05 | 1.87E-05 | 6.77E-07 | 0 | 0 | 3.19E-05 | 0 | 0 |
| sul1 | 0.000450936 | 8.82E-06 | 4.98E-06 | 5.90E-05 | 2.46E-05 | 3.88E-05 | 0.000141708 | 1.91E-05 | 4.58E-07 | 4.73E-05 | 2.03E-05 | 2.06E-05 | 2.06E-05 | 4.29E-06 | 1.84E-05 | 1.81E-06 | 0 | 1.48E-06 |
| sul2 | 6.39E-05 | 1.97E-05 | 0 | 0.000223881 | 0 | 1.41E-05 | 4.56E-05 | 8.98E-05 | 3.02E-06 | 1.71E-05 | 2.89E-06 | 0 | 1.93E-05 | 0 | 2.76E-06 | 4.05E-06 | 2.80E-06 | 0 |
| tet | 0 | 0 | 0 | 0 | 0 | 0 | 0 | 0 | 0 | 0 | 0 | 9.26E-07 | 0 | 0 | 0 | 0 | 0 | 0 |
| tet30 | 0 | 0 | 0 | 0 | 0 | 0 | 0 | 0 | 0 | 6.82E-06 | 4.09E-06 | 2.98E-06 | 0 | 0 | 6.97E-07 | 0 | 0 | 0 |
| tet31 | 0 | 0 | 0 | 0 | 0 | 0 | 0 | 0 | 0 | 4.68E-07 | 0 | 0 | 0 | 0 | 0 | 0 | 0 | 0 |
| tet32 | 4.34E-05 | 0 | 1.45E-06 | 0 | 4.60E-06 | 0 | 0 | 1.78E-07 | 1.00E-07 | 0 | 0 | 0 | 0 | 0 | 0 | 0 | 0 | 0 |
| tet33 | 0 | 0 | 0 | 0 | 0 | 6.66E-06 | 0 | 0 | 3.14E-07 | 0 | 1.13E-06 | 0 | 0 | 0 | 0 | 0 | 0 | 0 |
| tet34 | 0 | 5.53E-05 | 0 | 8.23E-05 | 0 | 0 | 0 | 8.06E-05 | 0 | 0 | 0 | 0 | 5.63E-06 | 0 | 0 | 0 | 0 | 0 |
| tet36 | 0 | 0 | 0 | 0 | 0 | 0 | 0 | 0 | 0 | 0 | 0 | 0 | 5.45E-07 | 0 | 0 | 0 | 0 | 0 |
| tet37 | 0 | 0 | 0 | 0 | 0 | 0 | 0 | 0 | 0 | 0 | 0 | 0 | 0 | 0 | 6.63E-07 | 0 | 0 | 0 |
| tet39 | 9.36E-05 | 2.16E-05 | 1.64E-05 | 2.15E-05 | 0.000450066 | 7.89E-05 | 0.000150296 | 0 | 6.32E-06 | 0.000521706 | 2.51E-05 | 9.37E-05 | 3.75E-05 | 0 | 0.000179256 | 5.19E-05 | 0 | 3.89E-05 |
| tet40 | 0 | 0 | 2.28E-06 | 0 | 0 | 0 | 0 | 2.80E-07 | 0 | 4.73E-07 | 0 | 0 | 8.45E-07 | 0 | 0 | 0 | 0 | 0 |
| tet41 | 0 | 0 | 0 | 0 | 0 | 0 | 0 | 0 | 0 | 0 | 0 | 0 | 0 | 0 | 0 | 0 | 0 | 1.62E-06 |
| teta | 0.001155765 | 0 | 2.33E-06 | 2.13E-05 | 0 | 6.81E-06 | 6.63E-05 | 0 | 9.44E-07 | 3.78E-05 | 4.59E-06 | 1.02E-05 | 3.22E-05 | 0 | 8.83E-06 | 0 | 0 | 1.06E-06 |
| tetb | 0 | 0 | 0 | 0 | 7.33E-06 | 0 | 0 | 0 | 0 | 1.43E-06 | 0 | 0 | 0 | 0 | 0 | 0 | 0 | 0 |
| tetc | 0 | 0.000182931 | 4.69E-05 | 8.16E-06 | 3.21E-05 | 6.06E-05 | 9.31E-05 | 0 | 1.64E-06 | 3.57E-05 | 3.90E-05 | 6.11E-05 | 9.15E-06 | 1.54E-06 | 3.42E-05 | 0 | 0 | 0.000517699 |
| tetd | 0 | 0 | 0 | 0 | 0 | 0 | 0 | 0 | 1.62E-07 | 2.44E-07 | 1.17E-06 | 0 | 0 | 0 | 0 | 0 | 0 | 0 |
| tete | 9.13E-05 | 0 | 1.14E-06 | 1.57E-05 | 0 | 1.00E-05 | 1.63E-05 | 2.36E-05 | 3.16E-07 | 3.77E-05 | 7.39E-06 | 1.45E-05 | 5.72E-05 | 0 | 4.07E-06 | 0 | 0 | 1.57E-06 |
| tetg | 0 | 0 | 0 | 0 | 4.25E-05 | 0 | 0 | 0 | 3.41E-07 | 2.80E-06 | 0 | 0 | 9.29E-07 | 0 | 0 | 0 | 0 | 0 |
| teth | 0.000235807 | 0 | 7.09E-06 | 2.69E-05 | 0.000202165 | 6.21E-05 | 0 | 8.71E-06 | 7.82E-06 | 1.71E-06 | 3.99E-05 | 7.50E-06 | 1.06E-05 | 0 | 2.80E-06 | 0 | 0 | 1.62E-06 |
| tetj | 0 | 0 | 9.31E-06 | 0 | 0 | 0 | 6.63E-05 | 0 | 0 | 0 | 0 | 0 | 0 | 0 | 0 | 0 | 0 | 0 |
| tetk | 0 | 0 | 3.74E-05 | 9.24E-06 | 7.68E-05 | 0 | 0.000460038 | 0 | 9.55E-05 | 1.26E-06 | 4.01E-06 | 5.12E-06 | 1.52E-06 | 3.92E-06 | 0 | 0 | 0 | 0 |
| tetl | 0 | 6.85E-05 | 0.001339835 | 5.53E-05 | 0.000102536 | 2.96E-06 | 0.000187298 | 0 | 0.000134483 | 0 | 3.01E-06 | 7.70E-06 | 0.00013945 | 0 | 2.40E-06 | 0 | 3.89E-06 | 2.31E-06 |
| tetm | 8.69E-05 | 0 | 0.00132674 | 1.33E-05 | 0.000296765 | 0 | 5.17E-05 | 1.78E-07 | 4.81E-06 | 2.70E-06 | 7.21E-07 | 5.52E-06 | 2.26E-05 | 0 | 0 | 0 | 0 | 0 |
| teto | 2.90E-05 | 0 | 0.000112494 | 0 | 0 | 0 | 0 | 0 | 1.00E-06 | 6.02E-07 | 0 | 9.21E-07 | 1.64E-06 | 0 | 0 | 0 | 0 | 0 |
| tetpa | 0 | 0 | 0 | 0 | 0 | 0 | 0 | 0 | 0 | 0 | 0 | 0 | 0 | 2.85E-06 | 0 | 0 | 0 | 0 |
| tetpb | 2.84E-05 | 0 | 0 | 0 | 0 | 0 | 0 | 0 | 0 | 0 | 0 | 0 | 0 | 0 | 0 | 0 | 0 | 0 |
| tetq | 0 | 0 | 8.66E-06 | 0 | 0 | 0 | 0 | 0 | 1.10E-06 | 2.93E-07 | 2.88E-06 | 0 | 4.60E-06 | 0 | 0 | 1.18E-05 | 0 | 0 |
| tets | 0.000201643 | 0.000293467 | 9.18E-05 | 3.64E-05 | 4.58E-06 | 1.27E-05 | 0.00012337 | 8.88E-07 | 1.10E-05 | 9.00E-07 | 4.89E-05 | 0.000276028 | 3.02E-05 | 1.86E-06 | 6.29E-05 | 0 | 0 | 3.30E-07 |
| tett | 0 | 0 | 0 | 0 | 0 | 0 | 0 | 0 | 1.97E-07 | 0 | 0 | 0 | 0 | 0 | 0 | 0 | 0 | 0 |
| tetu | 0 | 0 | 0 | 0 | 0 | 0 | 0 | 0 | 0 | 1.28E-05 | 0 | 0 | 0 | 0 | 0 | 0 | 0 | 0 |
| tetw | 5.79E-05 | 0 | 7.98E-06 | 0 | 0 | 0 | 2.07E-05 | 1.78E-07 | 0 | 0 | 0 | 0 | 2.46E-06 | 0 | 0 | 5.50E-06 | 0 | 0 |
| tetx | 0 | 0 | 8.36E-06 | 0 | 1.51E-05 | 6.98E-06 | 0 | 0 | 3.14E-06 | 1.26E-06 | 3.57E-06 | 0 | 8.98E-07 | 0 | 2.84E-06 | 0 | 0 | 3.28E-06 |
| tety | 9.46E-05 | 0 | 0 | 0 | 0 | 0 | 0 | 0 | 1.64E-07 | 2.46E-07 | 2.94E-06 | 0 | 8.91E-07 | 0 | 7.02E-07 | 0 | 0 | 5.42E-07 |
| tmrb | 0 | 0 | 0 | 0 | 0 | 0 | 0 | 0 | 0 | 0 | 2.32E-06 | 0 | 0 | 0 | 0 | 0 | 0 | 0 |
| tolc | 0 | 0.000337088 | 0.00016769 | 1.73E-05 | 7.76E-05 | 0.000164723 | 0.000161083 | 0 | 2.47E-06 | 0.000116037 | 0.000187053 | 0.000248707 | 2.97E-05 | 3.65E-06 | 0.000137954 | 1.07E-05 | 9.05E-06 | 0.001039352 |
| vanc | 0 | 0 | 0 | 0 | 0 | 0 | 0 | 0 | 0 | 0 | 2.68E-06 | 0 | 0 | 0 | 0 | 0 | 0 | 0 |
| vanrb | 0 | 0 | 0 | 0 | 0 | 0 | 0 | 0 | 0 | 0 | 4.18E-06 | 0 | 0 | 0 | 0 | 0 | 0 | 0 |
| vanrc | 0 | 0 | 0 | 0 | 0 | 0 | 0 | 0 | 0 | 0 | 3.98E-06 | 0 | 1.50E-06 | 0 | 2.37E-06 | 0 | 0 | 0 |
| vanrg | 0 | 0 | 0 | 0 | 0 | 0 | 2.80E-05 | 0 | 0 | 0 | 0 | 0 | 0 | 0 | 0 | 0 | 0 | 0 |
| vansc | 0 | 0 | 0 | 0 | 0 | 0 | 0 | 0 | 0 | 0 | 1.31E-06 | 0 | 4.95E-07 | 0 | 0 | 0 | 0 | 0 |
| vant | 5.30E-05 | 0 | 0 | 0 | 0 | 0 | 0 | 0 | 0 | 0 | 2.64E-06 | 5.06E-06 | 0 | 0 | 7.87E-07 | 0 | 0 | 0 |
| vanxyc | 0 | 0 | 0 | 0 | 0 | 0 | 0 | 0 | 0 | 0 | 2.42E-06 | 0 | 0 | 0 | 0 | 0 | 0 | 0 |
| vate | 0 | 0 | 0 | 0 | 0 | 0 | 0 | 0 | 0 | 0 | 0 | 0 | 8.12E-07 | 0 | 0 | 0 | 0 | 0 |
| ykkc | 0 | 0 | 8.22E-06 | 0 | 0 | 0 | 0 | 0 | 0 | 0 | 1.02E-05 | 0 | 0 | 0 | 0 | 0 | 0 | 0 |
| ykkd | 0 | 0 | 0 | 0 | 0 | 0 | 0 | 0 | 0 | 0 | 6.60E-06 | 0 | 0 | 0 | 0 | 0 | 0 | 0 |

^a^RTE, ready-to-eat

**Table. S6.** Detected antibiotic resistant gene types and corresponding abundances in these ready-to-eat food samples

| type | ^a^RTE meat.1 | RTE meat.2 | RTE meat.3 | RTE meat.4 | RTE meat.5 | RTE meat.6 | RTE meat.7 | RTE meat.8 | RTE vegetables.1 | RTE vegetables.2 | RTE vegetables.3 | RTE vegetables.4 | RTE vegetables.5 | RTE vegetables.6 | RTE vegetables.7 | RTE fruit.1 | RTE fruit.2 | RTE fruit.3 |
| --- | --- | --- | --- | --- | --- | --- | --- | --- | --- | --- | --- | --- | --- | --- | --- | --- | --- | --- |
| Multidrug | 0.036669043 | 0.490202139 | 0.113335074 | 0.143345862 | 0.257482746 | 0.439983726 | 0.357903414 | 0.152399219 | 0.055831878 | 0.378259238 | 0.426410232 | 0.421342827 | 0.502784042 | 0.331785099 | 0.528824692 | 0.302199427 | 0.476457476 | 0.575929287 |
| Unknown | 0.00064798 | 0.057077835 | 0.020799911 | 0.026737688 | 0.038470052 | 0.085092211 | 0.05099321 | 0 | 0.030253709 | 0.038488231 | 0.073390292 | 0.091257667 | 0.013602938 | 0.023289848 | 0.063204947 | 0.009980993 | 0.050694157 | 0.10285501 |
| Bacitracin | 0.015077994 | 0.082125738 | 0.076343521 | 0.142187038 | 0.125217397 | 0.061071445 | 0.043479042 | 0.03516307 | 0.200235617 | 0.106981341 | 0.091311068 | 0.15615656 | 0.254839738 | 0.252332948 | 0.129763796 | 0.061678611 | 0.132996364 | 0.089299009 |
| Aminoglycoside | 0.03202956 | 0.118913562 | 0.200403878 | 0.025896969 | 0.080926368 | 0.189807853 | 0.085111458 | 0.03125863 | 0.074349149 | 0.115010816 | 0.144093775 | 0.061986722 | 0.047116011 | 0.021990963 | 0.078339099 | 0.364062724 | 0.101665584 | 0.081359312 |
| Tetracycline | 0.059097276 | 0.122488209 | 0.244543726 | 0.236066141 | 0.397423573 | 0.076845683 | 0.175092132 | 0.196552051 | 0.358881476 | 0.200803443 | 0.056096049 | 0.089976948 | 0.087608032 | 0.039356031 | 0.075864286 | 0.155309253 | 0.014699077 | 0.044070442 |
| Fosmidomycin | 0.001110509 | 0.039916829 | 0.006186823 | 0.010629905 | 0.019581318 | 0.027953418 | 0.014488096 | 0 | 0.002083635 | 0.024780174 | 0.028846559 | 0.040347119 | 0.011775739 | 0.065166479 | 0.021509161 | 0.007013271 | 0.020251093 | 0.034469855 |
| Polymyxin | 0 | 0.023842381 | 0.00392592 | 0.005233511 | 0.008608173 | 0.016569725 | 0.004253991 | 0.000148003 | 0.002068389 | 0.014994992 | 0.020079627 | 0.01880218 | 0.020183769 | 0.014076753 | 0.01698351 | 0.003984676 | 0.030621224 | 0.030400949 |
| Beta-lactam | 0.050959754 | 0.017342392 | 0.019186033 | 0.026737095 | 0.021322656 | 0.048449888 | 0.045401643 | 0.00095726 | 0.018895052 | 0.07359226 | 0.048839049 | 0.039056777 | 0.010586989 | 0.035405618 | 0.025360601 | 0.018127847 | 0.067815358 | 0.028906111 |
| ^b^MLS | 0.005128565 | 0.004594698 | 0.235042809 | 0.042708038 | 0 | 0 | 0.085618109 | 0 | 0.238171424 | 0.001500854 | 0.051186887 | 0.014088701 | 0.01505542 | 0.186669037 | 0.004276349 | 0 | 0 | 0.006400273 |
| Fosfomycin | 0 | 0.007927472 | 0.003950587 | 0 | 0 | 0.005933146 | 0.026402704 | 0 | 0.001813885 | 0.005714672 | 0.022278917 | 0.003070976 | 0.000600345 | 0 | 0.003437263 | 0.046370971 | 0.047513214 | 0.004404212 |
| Quinolone | 0.016528254 | 0.021611298 | 0.075303079 | 0.020539072 | 0.027943724 | 0.026629652 | 0.040108843 | 0.243322274 | 0.000662414 | 0.010924884 | 0.020801505 | 0.04816993 | 0.02013945 | 0.01331163 | 0.044918846 | 0.008370217 | 0.031093642 | 0.001364684 |
| Chloramphenicol | 0.766907919 | 0.008338526 | 0.000575683 | 0.089686879 | 0.008906309 | 0.005269648 | 0.016122272 | 0.150972112 | 0.009564557 | 0.006082932 | 0.003497345 | 0.007514787 | 0.003470703 | 0 | 0.001353527 | 0.009755078 | 0.015617769 | 0.000397974 |
| Sulfonamide | 0.014363636 | 0.005618921 | 0.000402955 | 0.230231802 | 0.007919426 | 0.016393605 | 0.026549024 | 0.186718008 | 0.004628399 | 0.019410978 | 0.006915407 | 0.003820476 | 0.009728259 | 0.016615596 | 0.00536332 | 0.01314693 | 0.010575041 | 0.000114798 |
| Methicillin | 0 | 0 | 0 | 0 | 0 | 0 | 0 | 0 | 0 | 0 | 0 | 0 | 0 | 0 | 0 | 0 | 0 | 2.81E-05 |
| Vancomycin | 0.001479509 | 0 | 0 | 0 | 0 | 0 | 0.003971593 | 0 | 0 | 0 | 0.00512686 | 0.000935791 | 0.000488202 | 0 | 0.000800604 | 0 | 0 | 0 |
| Trimethoprim | 0 | 0 | 0 | 0 | 0.00619826 | 0 | 0.02450447 | 0.002509372 | 0 | 0.003455185 | 0.000435254 | 0.003472538 | 0.001384142 | 0 | 0 | 0 | 0 | 0 |
| Bleomycin | 0 | 0 | 0 | 0 | 0 | 0 | 0 | 0 | 0.002560416 | 0 | 0 | 0 | 0.000636222 | 0 | 0 | 0 | 0 | 0 |
| Tunicamycin | 0 | 0 | 0 | 0 | 0 | 0 | 0 | 0 | 0 | 0 | 0.000691174 | 0 | 0 | 0 | 0 | 0 | 0 | 0 |

^a^RTE, ready-to-eat; ^b^MLS, macrolide-lincosamide-streptogramin

**Table. S7.** Portion of representative antibiotic resistance genes subtypes in these ready-to-eat food samples

| ^a^ARG subtype | ^b^RTE meat | RTE vegetables | RTE fruit |
| --- | --- | --- | --- |
| *aac (6′) Ie* | 25.00% | 85.71% | 0.00% |
| *acrA* | 87.50% | 100.00% | 66.67% |
| *acrB* | 100.00% | 100.00% | 100.00% |
| *aph (3′)-IIIa* | 12.50% | 42.86% | 33.33% |
| *bacA* | 100.00% | 100.00% | 100.00% |
| *Bcr* | 87.50% | 100.00% | 100.00% |
| *Bl2e_cfxa* | 12.50% | 28.57% | 33.33% |
| *catA1* | 25.00% | 14.29% | 0.00% |
| *emrD* | 100.00% | 85.71% | 100.00% |
| *ermB* | 12.50% | 71.43% | 0.00% |
| *ermF* | 25.00% | 28.57% | 0.00% |
| *ksgA* | 75.00% | 100.00% | 100.00% |
| *lsa* | 12.50% | 14.29% | 0.00% |
| *macB* | 75.00% | 100.00% | 100.00% |
| *mdfA* | 62.50% | 100.00% | 100.00% |
| *mdtH* | 75.00% | 100.00% | 66.67% |
| *mexB* | 100.00% | 100.00% | 66.67% |
| *mexF* | 100.00% | 100.00% | 66.67% |
| *mexW* | 87.50% | 100.00% | 66.67% |
| *tet32* | 50.00% | 14.29% | 0.00% |
| *tet39* | 87.50% | 85.71% | 66.67% |
| *tet40* | 25.00% | 28.57% | 0.00% |
| *tetA* | 62.50% | 85.71% | 33.33% |
| *tetL* | 75.00% | 71.43% | 66.67% |
| *tetM* | 75.00% | 71.43% | 0.00% |
| *tetO* | 25.00% | 57.14% | 0.00% |
| *tetQ* | 12.50% | 57.14% | 33.33% |
| *tetW* | 50.00% | 14.29% | 33.33% |
| *tolC* | 75.00% | 100.00% | 100.00% |

^a^ARGs, antibiotic resistance genes; ^b^RTE, ready-to-eat

**Table. S8.** Summary of the shared antibiotic resistance genes among ready-to-eat meat, vegetables and fruit

| Sample introduction | Number of ^a^ARGs reference sequence | | |
| --- | --- | --- | --- |
|  | Total ARGs | Shared ARGs | The ratio of shared ARGs/ total ARGs (%) |
| ready-to-eat meat | 144 | 82 | 56.94 |
| ready-to-eat vegetables | 186 |  | 44.09 |
| ready-to-eat fruit | 98 |  | 83.67 |

^a^ARGs, antibiotic resistance genes

**Table. S9.** The diversity of microbial community and antibiotic resistance genes in these ready-to-eat food samples

| Sample name | ^a^OUT | ^b^Shannon index | Subtype | Reference sequence |
| --- | --- | --- | --- | --- |
| ^c^RTE meat.1 | 2303 | 6.84 | 32 | 766 |
| RTE meat.2 | 2622 | 7.1 | 37 | 868 |
| RTE meat.3 | 4003 | 5.63 | 53 | 1371 |
| RTE meat.4 | 1835 | 5.81 | 25 | 556 |
| RTE meat.5 | 4626 | 6.25 | 40 | 990 |
| RTE meat.6 | 2915 | 4.08 | 37 | 1006 |
| RTE meat.7 | 2500 | 6.59 | 47 | 1112 |
| RTE meat.8 | 3173 | 5.39 | 26 | 522 |
| RTE vegetables.1 | 3508 | 4.85 | 47 | 1280 |
| RTE vegetables.2 | 8388 | 3.18 | 56 | 1619 |
| RTE vegetables.3 | 12438 | 6.21 | 60 | 1707 |
| RTE vegetables.4 | 9927 | 6.48 | 53 | 1472 |
| RTE vegetables.5 | 10087 | 6.14 | 60 | 1505 |
| RTE vegetables.6 | 2252 | 4.12 | 26 | 661 |
| RTE vegetables.7 | 11055 | 4.94 | 46 | 1409 |
| RTE fruit.1 | 1783 | 2.67 | 32 | 656 |
| RTE fruit.2 | 2221 | 2.72 | 25 | 581 |
| RTE fruit.3 | 6783 | 5.39 | 49 | 1276 |

^a^OUT, operational taxonomic units; ^b^Shannon index , shannon index was calculated based on the OTU data; ^c^RTE, ready-to-eat

**Table. S10** Antibiotic resistance gene host information revealed by co-occurrence between antibiotic resistance genes subtype and microbial taxa

| Genus | ^a^ARG subtype | ARG type | Reference | Sample names in this study |
| --- | --- | --- | --- | --- |
| *Eubacterium_rectale*_group | *erm* | ^c^MLS | Not available | ^b^RTE meat.1, RTE meat.2, RTE meat.3, RTE meat.5, RTE meat.7, RTE meat.8, RTE vegetables.1, RTE vegetables.2, RTE vegetables.3, RTE vegetables.4, RTE vegetables.5, RTE vegetables.6, RTE vegetables.7, RTE fruit.1, RTE fruit.3 |
| *Vibrio* | *norm* | Multidrug | Multidrug transport protein NorM from Vibrio cholerae simultaneously couples to sodium-and proton-motive force | RTE meat.1, RTE meat.2, RTE meat.3, RTE meat.4, RTE meat.5, RTE meat.6, RTE meat.7, RTE meat.8, RTE vegetables.1, RTE vegetables.2, RTE vegetables.3, RTE vegetables.4, RTE vegetables.5, RTE vegetables.6, RTE vegetables.7, RTE fruit.1, RTE fruit.2, RTE fruit.3 |
| *Enhydrobacter* | *rosab* | Fosmidomycin | Not available | RTE meat.1, RTE meat.2, RTE meat.3, RTE meat.4, RTE meat.5, RTE meat.6, RTE meat.7, RTE meat.8, RTE vegetables.1, RTE vegetables.2, RTE vegetables.3, RTE vegetables.4, RTE vegetables.5, RTE vegetables.6, RTE vegetables.7, RTE fruit.1, RTE fruit.2, RTE fruit.3 |
| *Acinetobacter* | *adeabc* | Multidrug | AdeABC multidrug efflux pump is associated with decreased susceptibility to tigecycline in *Acinetobacter calcoaceticus*-*Acinetobacter baumannii* complex | RTE meat.1, RTE meat.2, RTE meat.3, RTE meat.4, RTE meat.5, RTE meat.6, RTE meat.7, RTE meat.8, RTE vegetables.1, RTE vegetables.2, RTE vegetables.3, RTE vegetables.4, RTE vegetables.5, RTE vegetables.6, RTE vegetables.7, RTE fruit.1, RTE fruit.2, RTE fruit.3 |
| *Klebsiella* | *mdtg* | Multidrug | Whole genome of *Klebsiella aerogenes* PX01 isolated from San Jacinto River sediment west of Baytown, Texas reveals the presence of multiple antibiotic resistance determinants and mobile genetic elements | RTE meat.1, RTE meat.2, RTE meat.3, RTE meat.4, RTE meat.5, RTE meat.6, RTE meat.7, RTE meat.8, RTE vegetables.1, RTE vegetables.2, RTE vegetables.3, RTE vegetables.4, RTE vegetables.5, RTE vegetables.6, RTE vegetables.7, RTE fruit.1, RTE fruit.2, RTE fruit.3 |
|  | *mdth* | Multidrug | Draft genome sequence of tetracycline-resistant *Klebsiella oxytoca* CCTCC M207023 producing 2,3-butanediol isolated from China |  |
|  | *mdtl* | Multidrug | Whole genome of *Klebsiella aerogene*s PX01 isolated from San Jacinto River sediment west of Baytown, Texas reveals the presence of multiple antibiotic resistance determinants and mobile genetic elements |  |
|  | *bcr_mfs* | Unknown | Not available |  |
|  | *rosab* | Fosmidomycin | Not available |  |
|  | *ksga* | Aminoglycoside | Draft genome sequence of a *Klebsiella pneumoniae* strain (new sequence type 2357) carrying Tn3926 |  |
|  | *acr* | Multidrug | Antibiotic resistance patterns and genetic analysis of *Klebsiella pneumoniae* isolates from the respiratory tract |  |
|  | *macab* | Multidrug | Resistance determinants and mobile genetic elements of an NDM-1-encoding *Klebsiella pneumoniae* strain |  |
|  | *emrd* | Multidrug | Investigation of 15 kinds of drug efflux genes in multidrug-resistant *Klebsiella pneumonia* |  |
|  | *mdtk* | Multidrug | Molecular typing and virulence analysis of multidrug resistant *Klebsiella pneumoniae* clinical isolates recovered from Egyptian hospitals |  |
| *Empedobacter* | *acr* | Multidrug | Not available | RTE meat.1, RTE meat.2, RTE meat.3, RTE meat.4, RTE meat.5, RTE meat.6, RTE meat.7, RTE meat.8, RTE vegetables.1, RTE vegetables.2, RTE vegetables.3, RTE vegetables.4, RTE vegetables.5, RTE vegetables.6, RTE vegetables.7, RTE fruit.2, RTE fruit.3 |
|  | *bcr_mfs* | Unknown | Not available |  |
|  | *mdtk* | Multidrug | Not available |  |
|  | *emrd* | Multidrug | Not available |  |
| *Pseudomonas* | *mexef* | Multidrug | Overexpression of the MexEF-OprN multidrug efflux system affects cell-to-cell signalling in *Pseudomonas aeruginosa* | RTE meat.1, RTE meat.2, RTE meat.3, RTE meat.4, RTE meat.5, RTE meat.6, RTE meat.7, RTE meat.8, RTE vegetables.1, RTE vegetables.2, RTE vegetables.3, RTE vegetables.4, RTE vegetables.5, RTE vegetables.6, RTE vegetables.7, RTE fruit.1, RTE fruit.2, RTE fruit.3 |
|  | *mexvw* | Multidrug | A new member of the tripartite multidrug efflux pumps, MexVW-OprM, in *Pseudomonas aeruginosa* |  |
|  | *mexab* | Multidrug | Influence of the MexAB-OprM multidrug efflux system on quorum sensing in *Pseudomonas aeruginosa* |  |
| *Enterobacter* | *mdtk* | Multidrug | Not available | RTE meat.1, RTE meat.2, RTE meat.3, RTE meat.4, RTE meat.5, RTE meat.6, RTE meat.7, RTE meat.8, RTE vegetables.1, RTE vegetables.2, RTE vegetables.3, RTE vegetables.4, RTE vegetables.5, RTE vegetables.6, RTE vegetables.7, RTE fruit.1, RTE fruit.2, RTE fruit.3 |
|  | *emrd* | Multidrug | Whole genome sequencing of *Klebsiella pneumoniae* strain unravels a new model for the development of extensive drug resistance in Enterobacteriaceae |  |
|  | *rosab* | Fosmidomycin | Not available |  |
|  | *bcr_mfs* | Unknown | Not available |  |
|  | *acr* | Multidrug | The AcrAB-TolC efflux pump contributes to multidrug resistance in the nosocomial pathogen *Enterobacter aerogenes* |  |
|  | *mdtg* | Multidrug | Not available |  |
|  | *mdtl* | Multidrug | Genome analysis of a wild rumen bacterium *Enterobacter aerogenes* LU2 - a novel bio-based succinic acid producer |  |
|  | *mdth* | Multidrug | Isolation of a strong promoter fragment from endophytic *Enterobacter cloacae* and verification of its promoter activity when its host strain colonizes banana plants |  |
|  | *macab* | Multidrug | Not available |  |
|  | *mdfa* | Multidrug | Biocide tolerance and antibiotic resistance of *Enterobacter spp.* Isolated from an Algerian hospital environment |  |
|  | *ksga* | Aminoglycoside | Not available |  |

^a^ARG, antibiotic resistance gene; ^b^RTE, ready-to-eat; ^c^MLS, macrolide-lincosamide-streptogramin

**Table. S11** Summary of target antibiotic resistance gene (ARG) detected using PCR in previous studies

| Food types | ARG types | ARG subtypes | Reference |
| --- | --- | --- | --- |
| raw and ready-to-eat retail food | Carbapenem | *bla-KPC* | Foodborne Klebsiella pneumoniae: virulence potential, antibiotic resistance, and risks to food safety |
| Ready to eat vegetables | Tetracycline | *tetA, tetB* | Detection of class 1 integron-associated gene cassettes and tetracycline resistance genes in Escherichia coli isolated from ready to eat vegetables. |
| fermented milk product | Vancomycin, Aminoglycoside,Tetracycline, Macrolide | *vanA*, *vanB*  *ant(6′)-Ia*, *aac(6′)-Ie-aph(2″)-Ia*, *aph(3″)-IIIa*  *tetM*, *tetL*  *ermB*, *ermA* | Ready-to-eat dairy products as a source of multidrug-resistant Enterococcus strains: Phenotypic and genotypic characteristics |
| ready-to-eat crickets | Tetracyclines, Vancomycin, β-lactams, Macrolide-lincosamide-streptogramin B, and Aminoglycosides | *tetM, tetO, tetK, tetS*  *ermA, ermB*, *ermC, blaZ , mecA*  *aac(6’)-Ie aph(2”)-Ia* | Investigating Antibiotic Resistance Genes in Marketed Ready-to-Eat Small Crickets (Acheta domesticus) |
| ready-to-eat seafood | Macrolide, Glycopeptide, Tetracycline | *ermA, vanA, tetA, tetM, eimB, vanB* | Antimicrobial Resistance, Virulence Determinants, and Biofilm Formation of Enterococcus Species From Ready-to-Eat Seafood |
| ready-to-eat grasshoppers and mealworms | Carbapenemase | *bla(NDM-1), bla(VIM), bla(GES), bla(OXA-48) bla(KPC))* | Investigation of the Dominant Microbiota in Ready-to-Eat Grasshoppers and Mealworms and Quantification of Carbapenem Resistance Genes by qPCR |
| ready-to-eat food samples | Aminoglycosides. Macrolide | *acc(6)/aph(2), aph(3)-III, ermA, ermB* | Molecular characteristics of antimicrobial resistance and virulence determinants of Staphylococcus aureus isolates derived from clinical infection and food |
| ready-to-eat meats | Meticillin, Tetracycline, macrolides and tetracyclines | *mecA, tetK,* *mphC, ermT* and *ermC* | Prevalence, toxigenic potential and antimicrobial susceptibility profile of Staphylococcus isolated from ready-to-eat meats |
| cheese samples and delicatessen meats | β-lactam | *blaTEM, blaSHV, blaOXY,blaCTX* | In vitro transference and molecular characterization of blaTEM genes in bacteria isolated from Portuguese ready-to-eat foods |
| ready-to-comsume deli and salads | Tetracycline | *tetL, tetS, tetK,tetM* | Tetracycline Resistance Associated with Commensal Bacteria from Representative Ready-to-Consume Deli and Restaurant Foods |
| fresh raw milk and cheese | Tetracycline, Macrolide | *tetS, tetM, tetA, ermB* | Food commensal microbes as a potentially important avenue in transmitting antibiotic resistance genes |
| live tilapia, crucian carp, catfish,and shrimp, | Tetracycline, Macrolide, Sulfonamide, β-lactam | *tetA, tetC, tetE, tetG ,tetS, tetL,teM, ermB, ermC, sul1 , sul2,blaTEM, blaCMY,blaCTX* | Antibiotic-Resistant Bacteria Associated with Retail Aquaculture Products from Guangzhou, China |
| artisanal raw milk cheese | Macrolide, Tetracycline | *ereA, ereB, mphA, ermA, ermB, ermC, mrsA,mrsB, mefA,mefE, tetM, tetL* | Enterococci from Appenzeller and Schabziger Raw Milk Cheese: Antibiotic Resistance, Virulence Factors, and Persistence of Particular Strains in the Products |
| cheeses,cured meats, sausages, smoked fishes, salads | Tetracycline, Macrolide | *tetL, tetK,tetM,mecA* | Retail Ready-to-Eat Food as a Potential Vehicle for Staphylococcus spp. Harboring Antibiotic Resistance Genes |
| retailed meat | Tetracycline,,Macrolide,Sulfonamide,Methicillin,β-lactam | *tetM, tetO, tetK, ermA, ermB, ermC, mecA, blaZ* | Isolation and Molecular Characterization of Antibiotic-Resistant Lactic Acid Bacteria from Poultry and Swine Meat Products |
| chicken salad, chicken burger, and carrot cake | Tetracycline, Macrolide, Aminoglycoside | *tetM,tetS,tetO,ermB,gelE,asal,esp,cylA* | Influx of Enterococci and Associated Antibiotic Resistance and Virulence Genes from Ready-To-Eat Food to the Human Digestive Tract |
| ready-to-eat food of animal origin (cheeses, cured meats, sausages, smoked fishes) | Macrolide, Tetracycline, Methicillin | *ermA, ermB, ermC, mrsA/B, tetK, tetL ,tetM, mecA* | Coagulase-negative staphylococci (CoNS) isolated from ready-to-eat food of animal origin – Phenotypic and genotypic antibiotic resistance |
| ready-to-eat dishes | Tetracycline, Macrolide, Aminoglycoside | *tetM, ermB, aac(6’)‐Ie‐aph(2”)* | Enterococci from ready-to-eat food horizontal gene transfer of antibiotic resistance genes and genotypic characterization by PCR Melting Profile |
| ready-to-eat meat product | Methicillin | *mecA* | Staphylococci isolated from ready-to-eat meat – Identification, antibiotic resistance and toxin gene profile |
| ready-to-eat salads | Tetracycline, Macrolide | *tetM, tetL and* *ermB* | Microbiological quality of ready-to-eat salads: An underestimated vehicle of bacteria and clinically relevant antibiotic resistance genes |
